# Healthcare professionals’ perspectives on and experiences with non-invasive prenatal testing: a systematic review

**DOI:** 10.1007/s00439-025-02736-y

**Published:** 2025-04-09

**Authors:** Chanelle Warton, Danya F. Vears

**Affiliations:** 1 Monash Bioethics Centre, Monash University, Melbourne, Australia; 2 Murdoch Children’s Research Institute, Melbourne, Australia; 3 Department of Paediatrics, University of Melbourne, Melbourne, Australia; 4 Centre for Biomedical Ethics and Law, KU Leuven, Leuven, Belgium; 5 School of Medicine, Deakin University, Geelong, Australia

## Abstract

Background: The increasing integration of non-invasive prenatal testing (NIPT) into antenatal practice and public healthcare systems globally raises both significant challenges in standardising service delivery and important ethical questions around routinisation and reproductive autonomy. This systematic review aims to synthesise existing primary empirical research on healthcare professionals’ views on and experiences with NIPT. Methods: A systematic search was conducted across four major databases in September 2023 and repeated in December 2024. Studies that reported findings from primary empirical research, including quantitative, qualitative and mixed methods research were included. Results: Searches returned 65 eligible articles, spanning 38 countries and 1 special administrative region and at least 12 professions. Views on who NIPT should have access to and which conditions should be screened for were influenced by perceived clinical utility. While healthcare professionals acknowledged NIPT as beneficial for supporting reproductive autonomy, concerns were raised about the amount and complexity of information to be conveyed during prenatal counseling and potential pressure to test. Cost was also identified as a significant barrier. Challenges reported during post-test counseling included communicating test failures and gaining information from laboratories. Views on the implications of NIPT for decision-making around abortion and for people with disabilities varied. Conclusions: Healthcare professionals play a critical role in facilitating the access to and decisions by pregnant people around prenatal genetic testing. Addressing barriers in clinical practice and increasing consistency across and access to clinical guidelines and education resources may support healthcare professionals in supporting reproductive autonomy.

**Supplementary Information:**

The online version contains supplementary material available at 10.1007/s00439-025-02736-y.

## Background

Non-invasive prenatal testing (NIPT) facilitates screening for fetal genetic conditions in early pregnancy by analysing cell-free DNA via a maternal blood sample to indicate the likelihood of a fetal genetic condition. Its use has become widespread since its introduction to clinical practice in 2011 and NIPT is now available in more than 60 countries (Ravitsky et al. 2021). Most commonly used to screen for common autosomal trisomies such as Trisomies 13, 18 and 21, the scope of NIPT has advanced to encompass expanded uses, including, but not limited to, sex chromosome aneuploidies, rare autosomal aneuploidies and microdeletions and duplications. However, the specificity and sensitivity of NIPT varies significantly depending on the condition screened (Jayashankar et al. 2023; Tian et al. 2023; Zaninović et al. 2022). The potential for the further development of the clinical use of NIPT to fetal genome-wide sequencing has been hypothesised in the academic literature and has been described by some as inevitable (Haidar et al. 2022; van Dijke et al. 2022). This rapid expansion of both the availability and scope of NIPT raises a number of practical, social and ethical challenges for the clinical practice of healthcare professionals, particularly for supporting the autonomous decision-making of pregnant people and their partners.

Reproductive autonomy, that is supporting pregnant people to make informed decisions about their pregnancies that align with their preferences and values, has been the prominent rationale behind the provision of prenatal testing in clinical and bioethical discourse (Ravitsky 2017). Healthcare professionals (HPs) play an integral role in autonomous reproductive decision-making about NIPT. However, HPs may experience challenges in ensuring the provision of sufficient information about NIPT and obtaining adequately informed consent, particularly in light of ongoing developments in test scope (Ravitsky 2017; Zaami et al. 2021). Inadequate counseling may result in decisional regret (i.e. post-test remorse following decision-making) and distress among people receiving unexpected or unwanted screening results (Ravitsky 2017; Zaami et al. 2021). While expanding the scope of NIPT has the potential to provide people with more information about their pregnancy, whether more information actually impedes the decision-making of prospective parents has also been questioned (Ravitsky 2017). Concerns have further been raised that pregnant people may experience an increased pressure to test from HPs who perceive there to be no reason not to test given the absence of miscarriage risk (Ravitsky 2017). While HPs can play a critical role in supporting pregnant people’s decision-making about NIPT, questions have been raised regarding whether adequate training and resources have been provided to HPs alongside the rapid diffusion of the test into clinical practice (Filoche et al. 2017; McKinn et al. 2022).

Healthcare professionals’ views and practices around offering NIPT have important implications in the decisions of pregnant people considering using this technology. Healthcare professionals also play a crucial role in facilitating the decision-making of pregnant people following test results (Jayashankar et al. 2023; Warton et al. 2023). Fourteen years after the clinical implementation of NIPT in 2011, an abundance of empirical research has been conducted on the views and experiences of HPs regarding the provision of NIPT globally. This paper aims to systematically review the primary empirical literature on this topic, providing insight into current challenges experienced by HPs who offer and counsel for NIPT as well as their views on emerging practical and ethical issues. 

## Methods

**Fig. 1:**
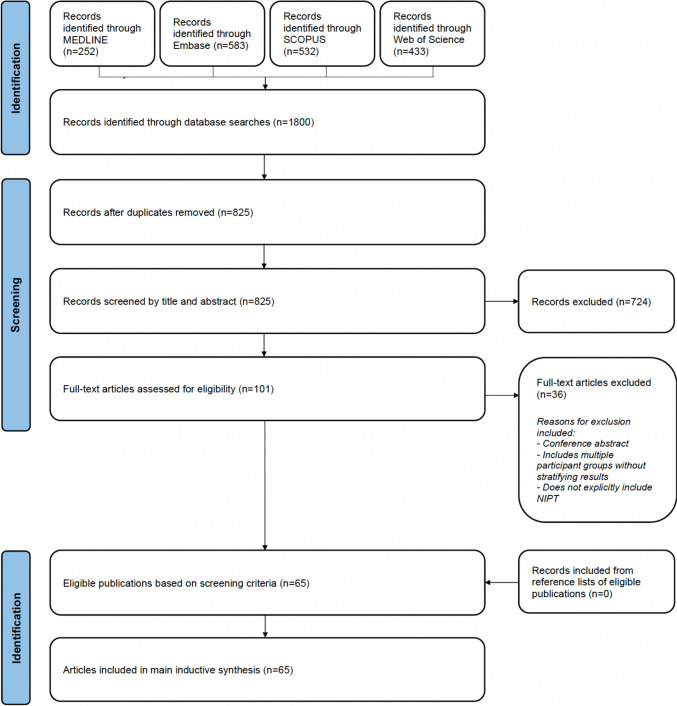
PRISMA diagram

Searches were conducted across MEDLINE, Embase, SCOPUS and Web of Science on 21 September 2023 and repeated on 5 December 2024 (Fig. 1). Search terms were developed to capture empirical literature focused on non-invasive prenatal testing (See Supplement 1). The protocol for this review was registered in PROSPERO (CRD42023421569).

Publications were included if they reported primary empirical research that explored HPs’ views on or experiences with prenatal testing, explicitly including NIPT. Literature was limited to research published in English. Results were excluded where they included participants other than HPs (such as public officials or pregnant people) and where stakeholders were not stratified or distinguishable. Publications were also excluded if they did not include the distinct discussion of NIPT, even if other reproductive technologies were discussed. No limitation was placed on publication year given the recent advent of NIPT into clinical practice and the aim to capture all relevant research to date, including those conducted prior to clinical implementation.

The titles and abstracts of database results were screened against inclusion criteria by CW and DV. All titles included by both researchers at this screening stage were progressed to full text screening by CW; any uncertainties were resolved through discussion. If titles were disputed at the abstract screening level (i.e. one researcher included and the other was unsure or excluded the title), the title underwent full-text screening. Quality appraisal tools were not utilised due to the diversity of studies and methodologies. Rather, a reason-based approach derived from bioethics was employed where content of articles were assessed during the data extraction process to critique and provide a comprehensive and unbiased overview of the information sought (Mertz 2019). This approach to quality appraisal is demonstrated in other systematic reviews of empirical genomics research (Vears et al. 2021).

Reference lists of articles included from initial database searches were also screened. Reference list titles were considered if they indicated an empirical study on HPs’ views about or experiences with prenatal genetic testing. These titles were then included subject to a full-text screen.

Inductive content analysis was employed to derive and analyse data from the included literature (Vears et al. 2021; Vears and Gillam 2022). Inductive content analysis is commonly used with text-based data, employing a close reading of texts to infer codes from the data as opposed to analysis using a pre-determined content list (Vears and Gillam 2022). Coding was an iterative process whereby data were initially coded using Word documents and subsequently uploaded to NVivo for final rounds of coding.

## Results

Our search identified 65 articles that met inclusion criteria (see Table 1), of which two thirds (64.6%, n = 42) of studies used quantitative research methods (see Table 2). Studies spanned research conducted across 38 countries and 1 special administrative region, with the greatest number of unique research studies conducted in the United States (n = 19, 29.2%), followed by the United Kingdom (n = 14, 21.5%), the Netherlands (n = 9, 13.8%) and Canada (n = 8, 12.3%). Data from a collective total of 9,971 participants was reported across all studies. Participants worked across at least 12 professions, primarily as obstetricians (OBs) and gynecologists (n = 3663, 36.7%), midwives (n = 2171, 21.8%), genetic counselors (GCs; n = 1838, 18.4%) and maternal fetal medicine specialists (MFMs; n = 721, 7.2%). Eleven key categories emerged from the literature: 1) how HPs offer NIPT; 2) views and experiences on the provision of pre-test counseling; 3) views and experiences on the provision of post-test counseling; 4) physician education and training; 5) the perceived association between NIPT and abortion; 6) views on genome-wide sequencing; 7) views on specific uses of NIPT; 8) perceived benefits and challenges for NIPT users; 9) perceived impact on people with disabilities; 10) cost and insurance and 11) views on regulation. Effort was made to reflect the language and scope used by HPs in a given article in the categorisation of results. The categories and sub-categories reported below are thus not necessarily mutually exclusive. For example, while microdeletions and microduplications may be considered part of expanded NIPT, they are categorised separately below to best reflect the language and scope conveyed in included literature.

**Table 1:** Publications included for analysis

Publication details				Study Details		
Title	Author	Year	Journal	Methodology	Country	Total HCP sample size
Supporting patient decision-making in non-invasive prenatal testing: A comparativestudy of professional values and practices in England and France	Bowman-Smart, Perrot & Horn	2024	BMC Medical Ethics	Qualitative	UK; France	35
Should non-invasive prenatal testing (NIPT) be used for fetal sex determination? Perspectives and experiences of healthcare professionals	Claesen- Bengton et al	2024	European Journal of Human Genetics	Qualitative	Belgium	23
Disparities in integrating non-invasive prenatal testing into antenatal healthcare in Australia: a survey of healthcare professionals	Johnston et al	2024	BMC Pregnancy and Childbirth	Quantitative	Australia	475
Challenges experienced by genetic counselors while they provided counseling about mosaic embryos	Moran et al	2023	Fertility & Sterility Reports	Quantitative	Canada; USA	78
Genetic counseling for fetal sex prediction by NIPT: Challenges and opportunities	Stevens et al	2023	Journal of Genetic Counseling	Quantitative	USA; Canada	147
Negotiating awareness: Dutch midwives’ experiences of noninvasive prenatal screening counseling	de Vries et al	2022	International Journal of Environmental Research and Public Health	Qualitative	Netherlands	8
Views of Canadian healthcare professionals on the future uses of non-invasive prenatal testing: a mixed method study	Haidar et al	2022	European Journal of Human Genetics	Mixed Methods	Canada	194
Clinician views and experiences of non-invasive prenatal genetic screening tests in Australia	McKinn et al	2022	Australian and New Zealand Journal of Obstetrics and Gynaecology	Qualitative	Australia	17
Health professionals and scientists’ views on genome-wide NIPT in the French public health system: Critical analysis of the ethical issues raised by prenatal genomics	Perrot & Horn	2022	PLOS ONE	Qualitative	France	17
Dynamics of reproductive genetic technologies: Perspectives of professional Stakeholders	van Dijke et al	2022	PLOS ONE	Qualitative	Netherlands	8
Access to prenatal testing and ethically informed counselling in Germany, Poland and Russia	Orzechowski et al	2021	Journal of Personalized Medicine	Qualitative	Germany; Poland; Russia	18
Understanding attitudes and behaviors towards cell-free DNA-based noninvasive prenatal testing (NIPT): A survey of European health-care providers	Benachi et al	2020	European Journal of Medical Genetics	Quantitative	France; UK; Italy; Spain; Germany	634
Implementing publicly funded noninvasive prenatal testing for fetal aneuploidy in Ontario, Canada: Clinician experiences with a disruptive technology	Burgess et al	2020	Qualitative Health Research	Qualitative	Canada	37
Implementation challenges for an ethical introduction of noninvasive prenatal testing: A qualitative study of healthcare professionals' views from Lebanon and Quebec	Haidar et al	2020	BMC Medical Ethics	Qualitative	Canada; Lebanon	20
Knowledge, attitude and practices of obstetricians and gynecologists on noninvasive prenatal testing with cell free fetal DNA in a private tertiary hospital	Panes & Javier	2020	Phillipine Journal of Obstetrics and Gynecology	Quantitative	Philippines	94
Implementation of high-throughput non-invasive prenatal testing for fetal RHD genotype testing in England: Results of a cross-sectional survey of maternity units and expert interviews	Ryczek, White & Carolan-Rees	2020	Transfusion Medicine	Mixed Methods	UK	25
Physicians' perception toward non-invasive prenatal testing through the eye of the Rogers' diffusion of innovation theory in China	Wei et al	2020	International Journal of Technology Assessment in Health Care	Quantitative	China	167
Prenatal screening in the era of non-invasive prenatal testing: A nationwide cross-sectional survey of obstetrician knowledge, attitudes and clinical practice	Yang & Tan	2020	BMC Pregnancy and Childbirth	Quantitative	Singapore	94
The value of non-invasive prenatal testing: Preferences of Canadian pregnant women, their partners, and health professionals regarding NIPT use and access	Birko et al	2019	BMC Pregnancy and Childbirth	Quantitative	Canada	184
Certified nurse-midwives' experiences with provision of prenatal genetic screening: A case for interprofessional collaboration	Dettwyler, Zielinski & Yashar	2019	Journal of Perinatal & Neonatal Nursing	Qualitative	USA	13
Canadian genetic healthcare professionals’ attitudes towards discussing private pay options with patients	Di Gioacchino, Langlois, & Elliott	2019	Molecular Genetics & Genomic Medicine	Quantitative	Canada	144
Current genetic counseling practice in the United States following positive non-invasive prenatal testing for sex chromosome abnormalities	Fleddermann et al	2019	Journal of Genetic Counseling	Quantitative	USA	176
Offering a choice between NIPT and invasive PND in prenatal genetic counseling: the impact of clinician characteristics on patients’ test uptake	van der Steen et al	2019	European Journal of Human Genetics	Qualitative	Netherlands	5
Genetic counselors’ perspectives about cell-free DNA: Experiences, challenges, and expectations for obstetricians	Agatisa et al	2018	Journal of Genetic Counseling	Qualitative	USA	25
Obstetrical provider knowledge and attitudes towards cell-free DNA screening: Results of a cross-sectional national survey	Chan et al	2018	BMC Pregnancy and Childbirth	Quantitative	Canada	202
Limits to the scope of non-invasive prenatal testing (NIPT): An analysis of the international ethical framework for prenatal screening and an interview study with Dutch professionals	Kater-Kuipers et al	2018	BMC Pregnancy and Childbirth	Qualitative	Netherlands	9
Preferences for prenatal testing among pregnant women, partners and health professionals	Lund et al	2018	Danish Medical Journal	Quantitative	Denmark	111
Introduction of non-invasive prenatal testing as a first-tier aneuploidy screening test: A survey among Dutch midwives about their role as counsellors	Martin et al	2018	Midwifery	Quantitative	Netherlands	436
Danish sonographers' experiences of the introduction of "moderate risk" in prenatal screening for Down syndrome	Møller et al	2018	Journal of Pregnancy	Qualitative	Denmark	5
Service provision of non-invasive prenatal testing for Down syndrome in public and private healthcare sectors: A qualitative study with obstetric providers	Ngan, Yi & Ahmed	2018	BMC Health Services Research	Qualitative	Hong Kong	20
Attitudes towards non-invasive prenatal diagnosis among obstetricians in Pakistan, a developing, Islamic country	Ahmed et al	2017	Prenatal Diagnosis	Quantitative	Pakistan	113
Evaluation of preferences of women and healthcare professionals in Singapore for implementation of noninvasive prenatal testing for Down syndrome	Barrett et al	2017	Singapore Medical Journal	Quantitative	Singapore	69
Survey of US obstetrician opinions regarding NIPT use in general practice: Implementation and barriers	Brewer, Demers & Musci	2017	Journal of Maternal–Fetal & Neonatal Medicine	Quantitative	USA	103
Views of the obstetric profession on non-invasive prenatal testing in Aotearoa New Zealand: A national survey	Filoche et al	2017	Australian and New Zealand Journal of Obstetrics and Gynaecology	Quantitative	New Zealand	134
Prenatal cfDNA screening results indicative of maternal neoplasm: survey of current practice and management needs	Giles et al	2017	Prenatal Diagnosis	Quantitative	USA	367
Preferences for prenatal diagnosis of sickle-cell disorder: A discrete choice experiment comparing potential service users and health-care providers	Hill et al	2017	Health Expectations	Quantitative	UK	62
Obstetric professionals' perceptions of non-invasive prenatal testing for Down syndrome: Clinical usefulness compared with existing tests and ethical implications	Ngan et al	2017	BMC Pregnancy and Childbirth	Quantitative	Hong Kong	327
Implementing non-invasive prenatal testing for aneuploidy in a national healthcare system: Global challenges and national solutions	Van Schendel et al	2017	BMC Health Services Research	Qualitative	Netherlands	6
Non-invasive prenatal diagnosis for BRCA mutations – A qualitative pilot study of health professionals’ views	Bennett, Chitty & Lewis	2016	Journal of Genetic Counseling	Qualitative	UK	6
Antenatal screening for aneuploidy-surveying the current situation and planning for non-invasive prenatal diagnosis in New Zealand	Eastwood et al	2016	New Zealand Medical Journal	Quantitative	New Zealand	108
The use of noninvasive prenatal testing in obstetric care: Educational resources, practice patterns, and barriers reported by a national sample of clinicians	Farrell et al	2016	Prenatal Diagnosis	Quantitative	USA	258
“I think we've got too many tests!”: Prenatal providers’ reflections on ethical and clinical challenges in the practice integration of cell-free DNA screening	Gammon et al	2016	Ethics, Medicine & Public Health	Qualitative	USA	21
Preferences for prenatal tests for Down syndrome: An international comparison of the views of pregnant women and health professionals	Hill et al	2016	European Journal of Human Genetics	Quantitative	Canada; Denmark; Iceland; Israel; Italy; Netherlands; Portugal; Singapore; UK	1245
Obstetrician and gynecologist utilization of the noninvasive prenatal testing expanded option	Mayes et al	2016	American Journal of Perinatology Reports	Quantitative	USA	85
The integration of noninvasive prenatal screening into the existing prenatal paradigm: A survey of current genetic counseling practice	Suskin et al	2016	Journal of Genetic Counseling	Quantitative	USA	208
Attitudes and knowledge of maternal–fetal medicine fellows regarding noninvasive prenatal testing	Swaney et al	2016	Journal of Genetic Counseling	Quantitative	USA	116
Survey of attitudes of Chinese perinatologists and obstetricians toward non-invasive prenatal genetic testing	Zhai et al	2016	Obstetrics and Gynaecology	Quantitative	China	392
Non-invasive prenatal testing: UK genetic counselors’ experiences and perspectives	Alexander, Kelly & Kerzin-Storrar	2015	Journal of Genetic Counselling	Qualitative	UK	20
Early clinical experience of cell-free DNA-based aneuploidy screening: A survey of obstetric sonologists in Australia and New Zealand	Hui et al	2015	Australian and New Zealand Journal of Obstetrics and Gynaecology	Quantitative	Australia; New Zealand	54
Global perspectives on clinical adoption of NIPT	Minear et al	2015	Prenatal Diagnosis	Quantitative	Argentina; Australia; Belgium; Canada; Chile; Cyprus; Czech Republic; France; Finland; Germany; Greece; Hong Kong; Hungary; India; Ireland; Israel; Italy; Mexico; Netherlands; Norway; Portugal; Qatar; Singapore; Sweden; Switzerland; Turkey; USA; UK	49
Changing to NIPT as a first-tier screening test and future perspectives: Opinions of health professionals	Tamminga et al	2015	Prenatal Diagnosis	Quantitative	Netherlands	240
Ethical concerns in the implementation of DNA sequencing-based noninvasive prenatal testing for fetal aneuploidy among obstetric professionals in Hong Kong	Yi et al	2015	AJOB Empirical Bioethics	Quantitative	Hong Kong	327
Obstetricians and gynecologists' practice and opinions of expanded carrier testing and noninvasive prenatal testing	Benn et al	2014	Prenatal Diagnosis	Quantitative	USA	222
NIPT: Current utilization and implications for the future of prenatal genetic counseling	Buchanan et al	2014	Prenatal Diagnosis	Quantitative	USA	202
Clinical implementation of noninvasive prenatal testing among maternal fetal medicine specialists	Haymon et al	2014	Prenatal Diagnosis	Quantitative	USA	278
Preferences for prenatal tests for cystic fibrosis: A discrete choice experiment to compare the views of adult patients, carriers of cystic fibrosis and health professionals	Hill et al	2014	Journal of Clinical Medicine	Quantitative	UK	70
Genetic counselors' experience with cell-free fetal DNA testing as a prenatal screening option for aneuploidy	Horsting et al	2014	Journal of Genetic Couneling	Quantitative	USA	236
Will the introduction of non-invasive prenatal testing for Down's syndrome undermine informed choice?	Silcock et al	2014	Health Expectations	Quantitative	UK	393
Views and preferences for the implementation of non-invasive prenatal diagnosis for single gene disorders from health professionals in the United Kingdom	Hill et al	2013	American Journal of Medical Genetics	Qualitative	UK	47
Non-invasive prenatal testing with cell-free DNA: US physician attitudes toward implementation in clinical practice	Musci et al	2013	Prenatal Diagnosis	Quantitative	USA	101
Determination of foetal sex in pregnancies at risk of haemophilia: A qualitative study exploring the clinical practices and attitudes of health professionals in the United Kingdom	Hill et al	2012	Haemophilia	Qualitative	UK	32
Women's and health professionals' preferences for prenatal tests for down syndrome: A discrete choice experiment to contrast noninvasive prenatal diagnosis with current invasive tests	Hill et al	2012	Genetics in Medicine	Quantitative	UK	181
Attitudes toward non-invasive prenatal diagnosis among pregnant women and health professionals in Japan	Yotsumoto et al	2012	Prenatal Diagnosis	Quantitative	Japan	185
Cell-free fetal DNA testing: A pilot study of obstetric healthcare provider attitudes toward clinical implementation	Sayres et al	2011	Prenatal Diagnosis	Quantitative	Not specified	62
Will the introduction of non-invasive prenatal diagnostic testing erode informed choices? An experimental study of health care professionals	van den Heuvel et al	2010	Patient Education and Counseling	Quantitative	UK	231

**Table 2:** Demographic information of included publications

Category	Number of Studies (n = 65)	Percentage of Studies	Number of Participants (n = 9971)	Percentage of Participants
*Research Methodology*				
Quantitative	42	64.6%	–	–
Qualitative	21	32.3%	–	–
Mixed-Methods	2	3.1%	–	–
*Country*				
USA	19	29.2%	2820	28.3%
UK	14	21.5%	1449	14.5%
Netherlands	9	13.8%	985	9.9%
Canada	8	12.3%	817	8.2%
Hong Kong	4	6.2%	679	6.8%
China	2	3.1%	559	5.6%
Australia	4	6.2%	547	5.5%
Italy	3	4.5%	251	2.5%
New Zealand	3	4.6%	244	2.4%
Singapore	4	6.2%	233	2.3%
Denmark	3	4.6%	225	2.3%
Japan	1	1.5%	185	1.9%
France	4	6.2%	158	1.6%
Germany	3	4.6%	139	1.4%
Spain	1	1.5%	130	1.3%
Pakistan	1	1.5%	113	1.1%
Israel	2	3.1%	99	1.0%
Philippines	1	1.5%	94	0.9%
Iceland	1	1.5%	61	0.6%
Portugal	2	3.1%	52	0.5%
Belgium	1	1.5%	24	0.2%
Lebanon	1	1.5%	10	0.1%
Other	1	1.5%	32	0.3%
Not specified	2	3.1%	65	0.7%
*Profession*				
Obstetrician*	53	81.5%	3663	36.7%
Midwife**	26	40.0%	2171	21.8%
Genetic counsellor	24	36.9%	1838	18.4%
MFM	9	13.8%	721	7.2%
Nurse**	16	24.6%	496	5.0%
GPs	11	16.9%	332	3.3%
Clinical / medical geneticist	17	26.2%	199	2.0%
Sonographer	5	7.7%	75	0.8%
Paediatrician	1	1.5%	39	0.4%
Haematologist	2	3.1%	14	0.1%
Other	15	23.1%	290	2.9%
Not specified	12	18.5%	133	1.3%

* Includes perinatologists as profession not stratified in Zhai et al.

** Includes certified nurse midwives from Dettwyler et al. and nurses from Yotsumoto et al. as profession not stratified

**Includes clinical geneticists as profession not stratified in Johnston et al.

### Offering NIPT

#### Self-reported factors influencing the decision to offer NIPT

Healthcare professionals across Europe, the USA, China and the Philippines reported their decision to offer NIPT was influenced by recommendations and guidelines of professional associations (Benachi et al. 2020; Haymon et al. 2014; Musci et al. 2013; Panes & Javier 2020; Sayres et al. 2011; Wei et al. 2020). Healthcare professionals in France were the exception, whose use of NIPT was not strongly influenced by local or international association guidelines (Benachi et al. 2020). American OBs and MFMs additionally reported published clinical data and insurance coverage as factors influencing NIPT adoption (Haymon et al. 2014; Musci et al. 2013; Sayres et al. 2011). Insufficient published clinical data, cost or lack of insurance coverage and disinterest among pregnant people were similarly noted as factors by Filipino OBs (Panes & Javier 2020). Private genetic testing companies were also indicated to be a key reason for offering NIPT among Chinese OBs (Wei et al. 2020). Most New Zealand OBs reported their personal attitudes or beliefs about antenatal screening did or would not affect their decision to offer NIPT (74.%, n = 90) but that their knowledge about NIPT would (70.3%, n = 85) (Filoche et al. 2017).

American GCs in one study indicated that their institution decided whether or not NIPT would be offered. Perceived institutional reasons for offering NIPT included the view that all testing options should be available to all pregnant people, NIPT as a good alternative to invasive testing, perceived demand and the need to stay competitive (Horsting et al. 2014). A study of NIPT use among Chinese OBs similarly reported that NIPT adoption by other physicians and potential hospital competition might increase test uptake (Wei et al. 2020).

#### Indications for offering NIPT

Healthcare professionals’ views and practices on to whom should be offered NIPT were varied. Most HPs were supportive of offering NIPT to high risk individuals (Alexander et al. 2015). Nearly all (94.1%) of American GCs (Buchanan et al. 2014) and half (45%) of Filipino OBs (Panes & Javier 2020). offered NIPT to pregnant people they considered to be high risk. European healthcare providers reported consistently offering (78–95%) or actively recommending (62%-90%) NIPT to people with high risk pregnancies (Benachi et al. 2020). High risk indicators included advanced maternal age, abnormal ultrasound findings, positive maternal serum screening (MSS), repeated spontaneous abortion and a personal or family history of chromosome abnormalities (Benachi et al. 2020; Buchanan et al. 2014; Filoche et al. 2017; Haymon et al. 2014; Horsting et al. 2014; Zhai et al. 2016). Nearly half (46.3%, n = 19) of the HPs in one multi-country study reported offering NIPT exclusively to people with high risk pregnancies (Minear et al. 2015) compared to just 11.9% of American OBs who believed NIPT should be exclusively offered to those who were high risk (Musci et al. 2013). British GCs expressed the view that high risk pregnancies among those with a family history of a condition should be provided NIPT in a genetics setting, most (60%, n = 12) believed increasingly routine use of NIPT would inevitably mean the responsibility of offering testing would fall to midwives due to genetic counseling resource constraints (Alexander et al. 2015). Some (15%) American and Canadian GCs recommended NIPT following a mosaic embryo transfer (Moran et al. 2023). American GCs (73%) also noted that NIPT was often offered when people with high risk pregnancies declined invasive diagnostic testing (Haymon et al. 2014).

While the majority (71.8%) of American OBs in 2017 supported offering NIPT to all pregnant people regardless of age or risk status (Brewer et al. 2017). More than half (55.4%) of Dutch midwives in a 2017 study similarly felt it was unacceptable not to offer NIPT to everyone (Martin et al. 2018). In a 2012 study, over a third (35.9%) of Japanese HPs believed that NIPT should be available to all pregnant people (Yotsumoto et al. 2012). A fifth (19.5%, n = 8) of HPs similarly supported universal access in a 2015 global study (Minear et al. 2015). In a 2016 survey of American GCs, 44.7% (n = 85) supported universal access but only 11.5% (n = 22) offered it to all who presented for prenatal care (Suskin et al. 2016). Rates of offering NIPT to all pregnant women were significantly lower than rates of support for universal access. In 2014, approximately 5% of American MFMs (Haymon et al. 2014) and between 1.7% to 3.9% of American GCs offered NIPT to all pregnant people (Buchanan et al. 2014; Horsting et al. 2014). When asked whether professional association guidelines should be updated to include NIPT as a screening option for pregnant people of any risk status in 2017, 87.4% of American OBs supported this (Brewer et al. 2017). American GCs' reasons for supporting universal access to NIPT included the perception that NIPT was superior to other available screening tests and satisfaction with test validation studies in low-risk populations (Suskin et al. 2016). The most common reason for offering NIPT to all amongst American OBs in 2017 was the detection of Down syndrome (Brewer et al. 2017). Common reasons for not providing NIPT to all pregnant people included lack of validation and lower test performance in low risk populations as well as insufficient provider education or understanding and lack of understanding among potential test users (Suskin et al. 2016).

Almost all OBs (96%) in a 2015 Australian and New Zealand study indicated they were willing to offer NIPT for low risk pregnancies (Hui et al. 2015). American GCs also reported offering NIPT to people with low risk pregnancies who presented too late for other screening tests or were unable to use other prenatal screens in 2016 (Suskin et al. 2016). Between 5 to 29% of European HPs in 2020 were likely to recommend NIPT for low risk pregnancies, with lowest percentages being in the UK and France and the highest in Germany and Italy (Benachi et al. 2020). Some American GCs in 2018 viewed the expansion of NIPT to low risk pregnancies to be problematic as it necessitated major changes in clinical practice, particularly in relation to pre and post-test counseling (Agatisa et al. 2018).

Requests by the pregnant person were also reported as an indicator for providing NIPT by some HPs. Almost all (93%, n = 109) OBs in a New Zealand study accounted for such requests when considering offering NIPT (Filoche et al. 2017). Almost half (55.3%) of American MFMs reported offering NIPT when specifically requested (Haymon et al. 2014). American GCs in a 2018 qualitative study reported that they considered a pregnant person’s autonomy to be the final determinant in offering NIPT and thus would support people with low risk pregnancies to access the test should they request it (Agatisa et al. 2018). More than a third (37.8%, n = 76) of GCs in another American study similarly would provide NIPT for people with low risk pregnancies at their request (Suskin et al. 2016). A minority of American GCs in a 2014 study (1.7%)(Buchanan et al. 2014) and HPs in a global study in 2015 (14.6%, n = 6) (Minear et al. 2015) reported offering NIPT only to those who requested it.

#### Use of NIPT in context of other prenatal tests

##### Viability scans

Viability scans are commonly undertaken at 6–10 weeks gestation to determine whether a pregnancy is developing normally. Viability scans are able to detect some circumstances in which a pregnancy would not be viable, such as tubal ectopic pregnancies or the absence of a fetal heart activity. Several Australian HPs in a 2020 study reported examples of NIPT being conducted without a viability scan, (McKinn et al. 2022) indicating the possibility of NIPT being undertaken for non-viable pregnancies.

#### NIPT as a first-line screening test

Many HPs reported that NIPT was already being used as a first-line screening test or that they believed it would soon be used as such (Birko et al. 2019; Horsting et al. 2014; Johnston et al. 2024). Dutch HPs thought replacing first trimester screening with NIPT would be advantageous, making the explanation of test procedures easier and simplifying pre-test counseling (Martin et al. 2018; Tamminga et al. 2015; Van Schendel et al. 2017). Other HPs believed that NIPT should or would replace MSS (Benachi et al. 2020; Haymon et al. 2014). Perceived advantages of NIPT over combined first trimester screening (CFTS) included increased accuracy, (Gammon et al. 2016) sensitivity and specificity (Agatisa et al. 2018). While many HPs in Hong Kong, Italy and the USA viewed NIPT as a first-line screening test, (Benachi et al. 2020; Brewer et al. 2017; Yi et al. 2015) other HPs in the USA and Europe did not typically offer NIPT as a first-line screen (Benachi et al. 2020; Suskin et al. 2016).

Many HPs emphasised the importance of continuing to offer an 11–13 week ultrasound alongside NIPT as a first-line screening test (Eastwood A. et al., 2016; Filoche et al. 2017; Hui et al. 2015; Martin et al. 2018; Suskin et al. 2016; Tamminga et al. 2015). Reasons for providing 11–13 week ultrasounds alongside NIPT included the detection of structural anomalies, prediction of adverse obstetric outcomes, such as pre-eclampsia, and parental reassurance (Hui et al. 2015). UK GCs in 2015 also reported reassurance for themselves arising from the use of an ultrasound (Alexander et al. 2015).

#### NIPT as a second-line screening test

Healthcare professionals in multiple studies considered offering NIPT as a second-line prenatal screening test as appropriate (Birko et al. 2019; Dettwyler et al. 2019; Martin et al. 2018). The use of NIPT as a second-line screen was viewed positively as it provided greater accuracy when compared to other screening tests but avoided the miscarriage risk associated with diagnostic testing (Dettwyler et al. 2019). Most (73%—95%) European HPs in a 2020 were likely to recommend NIPT following an abnormal screening result (Benachi et al. 2020). Many HPs in a 2020 study in Singapore indicated they would offer NIPT following positive first trimester screening results for Trisomy 21 (Yang & Tan 2020). Fewer (24.5%) HPs in Hong Kong viewed NIPT as a second-line screen (Yi et al. 2015). A minority (22%, n = 53) of Dutch HPs in 2015 thought that NIPT should be offered following high chance CFTS (Tamminga et al. 2015).

#### Diagnostic testing

Diagnostic testing was primarily offered to pregnant people following a positive NIPT result (Benachi et al. 2020; Haymon et al. 2014; Swaney et al. 2016). One Canadian study in 2018 found that MFMs (96.6%) and OBs (91.2%) were more likely to offer invasive testing following a positive NIPT result compared to GPs (77.4%) and midwives (78.6%) (Chan et al. 2018).

Some HPs viewed NIPT as a substitute for diagnostic testing, with 47.9% of American OBs in a 2014 study indicating they would consider NIPT as a complete substitute for invasive testing in instances of high chance CFTS results for Trisomy 21 (Benn et al. 2014). Approximately a quarter (27%) of HPs in Hong Kong in 2015 who provided NIPT similarly regarded it as a diagnostic test (Yi et al. 2015). Some (14.6%) American OBs in 2017 also believed NIPT to be a diagnostic test for fetal aneuploidy (Brewer et al. 2017). A minority (2.2%) of HPs in a 2019 Canadian study also considered it appropriate to use NIPT as a diagnostic test (Birko et al. 2019).

While most (74.2%) American MFMs did not believe NIPT should be used in place of diagnostic testing, many European (53–69%) and American (28%-47%) HPs agreed that NIPT would replace diagnostic testing in the future (Benachi et al. 2020; Haymon et al. 2014; Horsting et al. 2014). However, American GCs (20.3%, n = 42) (Horsting et al. 2014) did not believe NIPT would replace diagnostic testing.

#### Choice of NIPT provider

Harmony (Ariosa Diagnostics) was the brand of NIPT test most commonly used by HPs globally (Hui et al. 2015; Minear et al. 2015; Yang and Tan 2020). Other brands used included Panorama (Natera), (Hui et al. 2015; Minear et al. 2015; Yang and Tan 2020). iGene (BGI),(Hui et al. 2015; Minear et al. 2015; Yang & Tan 2020). Verifi (Illumina), (Hui et al. 2015; Minear et al. 2015). MaterniT21 Plus (Sequenom), (Hui et al. 2015; Minear et al. 2015) and PrenaTest (Lifecodexx) (Minear et al. 2015). More than half (65%) of New Zealand HPs did not know their NIPT provider (Eastwood A. et al., 2016) Cost, (Hui et al. 2015; Johnston et al. 2024; Yang and Tan 2020) perceived test quality or performance,(Hui et al. 2015; Johnston et al. 2024; Yang & Tan 2020) a pre-existing relationship with the laboratory(Hui et al. 2015), clinical support (Johnston et al. 2024; Yang & Tan 2020) and brand familiarity (Johnston et al. 2024) were the most influential factors in HPs’ choice of NIPT provider. Institutional availability and policies also affected which brands of NIPT HPs utilised (Hui et al. 2015; Yang & Tan 2020). Genetic counselors in one American study perceived cost and insurance considerations as the most influential factor in their institution’s choice of provider (Horsting et al. 2014). Several HPs noted that pregnant people choose their NIPT provider (Minear et al. 2015).

### Pre-test counseling

#### Supporting informed decision-making

Most HPs viewed pre-test counseling as an important and necessary part of the provision of prenatal testing (Alexander et al. 2015; Benachi et al. 2020; Haymon et al. 2014; Horsting et al. 2014; Kater-Kuipers et al. 2018; Sayres et al. 2011; Stevens et al., 2023). Healthcare professionals viewed pre-test counseling as supporting informed decision-making(Agatisa et al. 2018) and autonomous choice (van Dijke et al. 2022). Dutch midwives viewed the provision of value-free counseling that supported pregnant people to make decisions aligned with their personal values as supporting autonomous decision-making (de Vries et al. 2022). While some HPs emphasised the importance of non-directiveness, (Bowman-Smart et al. 2024; de Vries et al. 2022) others differed in their levels of non-directiveness during pre-test counseling (van der Steen et al. 2019). Healthcare professionals who emphasised non-directiveness described prenatal testing as a personal decision and stressed the need for value-neutral or value-free communication about testing options, (Bowman-Smart et al. 2024; de Vries et al. 2022) whereas others questioned whether value-neutrality was attainable, provided value judgements or reported instances of deciding with the pregnant person (van der Steen et al. 2019). A Danish study further demonstrated that HPs and women differ in the value they place on test attributes, with HPs emphasising test accuracy whereas women priortised the absence of miscarriage risk (Lund et al. 2018).

Practical limits on non-directive counseling were noted by some Dutch HPs, including the influence of personal communication style, non-verbal communication or alloting different durations to the explanation of each respective prenatal test, which may inadvertently convey value judgements (van der Steen et al. 2019).

While some Dutch HPs viewed autonomous choice as inclusive of the option for pregnant people to decline learning about NIPT, (Kater-Kuipers et al. 2018; van Dijke et al. 2022) others questioned whether pregnant people could be truly informed about what prenatal testing entails at the time of declining further information (Kater-Kuipers et al. 2018).

Despite the importance placed on pre-test counseling for supporting informed choice, some HPs raised concerns that NIPT was being provided without adequate counseling. A third of OBs and midwives in Hong Kong were concerned that NIPT could be performed without prior counseling (33%) and were concerned about pregnant people not having adequate information about NIPT (36%) (Yi et al. 2015). Examples of NIPT being conducted with limited pre-test counseling were reported by several HPs in one Australian study (McKinn et al. 2022).

#### Content of pre-test counseling

During pre-test counseling, HPs generally reported describing NIPT as a screening test, (Buchanan et al. 2014; Farrell et al. 2016; McKinn et al. 2022) presenting alternative testing options, (Buchanan et al. 2014; Dettwyler et al. 2019; Farrell et al. 2016; McKinn et al. 2022)discussing the conditions being screened for (Buchanan et al. 2014; Filoche et al. 2017; McKinn et al. 2022; Ngan et al. 2018) and test limitations (Filoche et al. 2017; McKinn et al. 2022; Ngan et al. 2018). Test limitations included noting test performance, (Farrell et al. 2016; Filoche et al. 2017) test sensitivity (Ngan et al. 2018) and the potential for false positives and negatives (Benachi et al. 2020; Buchanan et al. 2014). Healthcare professionals in New Zealand and the USA also discussed test cost and/or insurance coverage (Buchanan et al. 2014; Di Gioacchino et al. 2019; Filoche et al. 2017). Procedural aspects of NIPT, namely that NIPT is a blood test, were also discussed (Ngan et al. 2018).

While HPs noted it was essential for pregnant people to consider the potential implications of a positive result prior to testing, (de Vries et al. 2022) many felt that pregnant people were unlikely to give these due consideration(Ahmed et al. 2017) or that they assumed they would receive a negative result (de Vries et al. 2022). While many HPs recommended that any positive NIPT results be followed with diagnostic testing during pre-test counseling,(Buchanan et al. 2014; Dettwyler et al. 2019; Filoche et al. 2017) most (84%) of Dutch HPs did not believe discussions about diagnostic testing during pre-test counseling were necessary as few women would receive positive results (Tamminga et al. 2015). Approaches to discussing the implications of positive results varied. Approximately half (48.4%, n = 124) of American OBs and MFMs reported often or always discussed considerations of raising a child with a serious medical condition or disability or the option of termination (Farrell et al. 2016). In contrast, HPs in Hong Kong did not discuss the possibility of raising a child with a condition or disability or termination, indicating that these topics were more relevant to invasive diagnostic testing or were too sensitive to be raised at this stage (Ngan et al. 2018).

Some HPs additionally emphasised the potential for specific results. UK GCs who had professional experience receiving inconclusive test results reported placing greater emphasis on this possibility in subsequent consultations (Alexander et al. 2015). Seventy percent (70%, n = 90) of American and Canadian GCs disclosed the ability of NIPT to predict fetal sex during pre-testing counseling, whereas 6.3% (n = 8) indicated they would not unless explicitly asked by the pregnant person (Stevens et al. 2023).

The potential for insufficient pre-testing counseling arising from inadequate physician knowledge was raised in several studies (Agatisa et al. 2018; Burgess et al. 2020; Haidar et al. 2020; Yi et al. 2015). American GCs in 2018 reported a then-emerging problem of needing to spend greater time during counseling correcting misunderstanding about NIPT among test users, particularly about the indications for NIPT use, that the test was diagnostic and that its primary function was to provide information about fetal sex (Agatisa et al. 2018).

#### Obtaining consent

Methods of obtaining consent for NIPT varied. In a study of UK OBs and midwives conducted in 2010 prior to the clinical implementation of NIPT, HPs were less likely to perceive a need to sign a consent form for NIPT (68%) compared to invasive prenatal diagnosis (98%) (van den Heuvel A. et al., 2010). Most (62.2%) American GCs indicated they gained consent verbally in 2016, (Buchanan et al. 2014) whereas 57.8% (n = 70) New Zealand OBs required pregnant people to sign a written consent form (Filoche et al. 2017). Some American GCs also received consent in writing, either through an NIPT-specific consent form (29%) or an existing prenatal testing consent form which has been modified to incorporate NIPT (8%) (Buchanan et al. 2014). American GCs differed in their views on whether an NIPT-specific consent form was necessary, with 45.1% in support and 22.0% deeming it unnecessary (Buchanan et al. 2014). Some HPs in France and New Zealand also used laboratory consent or requisition forms (Filoche et al. 2017; Perrot & Horn 2022). French HPs reported modifying laboratory consent forms to request pregnant people explicitly refuse or consent to testing, as opposed to providing an opt-in only choice (Perrot & Horn 2022).

Healthcare professionals raised concerns about obtaining adequately informed consent for NIPT, (Alexander et al. 2015) noting that people may not fully consider possible implications of the test (Filoche et al. 2017; Tamminga et al. 2015). However, British GCs (60%, n = 12) noted similar challenges in achieving informed consent in existing clinical practice around prenatal screening for Trisomy 21 (Alexander et al. 2015). French HPs raised further concerns about the complexity of achieving consent when screening for conditions beyond common trisomies (Perrot & Horn 2022).

#### Professions responsible for offering NIPT

Most (91.4%, n = 213) American GCs in a 2014 study had offered NIPT (Horsting et al. 2014). In another American study from 2014, all GCs (100%, n = 202) indicated that they obtained consent for NIPT in their clinics and 31.8% indicated the MFMs were also obtaining consent for NIPT (Buchanan et al. 2014). In 2016, almost half (45.4%, n = 89) of American GCs indicated that all pregnant people receiving NIPT go through genetics (Suskin et al. 2016). In a 2023 study, the majority (67.7%) of American and Canadian GCs provided counseling to people with high risk pregnancies often or sometimes, with 26.2% counseling for high risk pregnancies very often (Stevens et al. 2023). American and Canadian GCs reported most referrals for NIPT coming from OBs (91.2%) or MFMS (61.2%) (Stevens et al. 2023). Approximately half of American OBs in a 2013 study reported managing screening tests for high risk pregnancies, whereas other referred to perinatology subspecialists (Musci et al. 2013). Most (68%) reported managing screening for people with average risk pregnancies themselves (Musci et al. 2013). In a 2016 study, most (82%, n = 69) American OBs indicated having ordered NIPT. Five per cent (5%, n = 4) reported always referring to an MFM or GC (Mayes et al. 2016). A 2017 study in New Zealand reported NIPT did not appear to be routinely offered, with 14.8% (n = 18) of OBs frequently and 38.5% (n = 47) sometimes offering the test (Filoche et al. 2017). In a 2020 study of European healthcare providers, familiarity with NIPT was lowest among midwives in the UK (2%) compared to those in other countries (17%-31%). This was reflected in referral practices where physicians were more likely to offer NIPT or refer out for it (75%-93%) than midwives (45%–82%) (Benachi et al. 2020).

Distinct from the actual provision of pre-test counseling, views on who *should* be responsible for the provision of pre-test counseling varied. In a 2014 survey of American GCs, most (97.7%) indicated that GCs should consent for NIPT, followed by MFMs (60.3%), OBs (9.8%), nurse practitioners (8.0%) and midwives (5.2%) (Buchanan et al. 2014). American GCs in 2014 at least somewhat disagreed that pre-testing counseling for NIPT could be administered by GCs and other HPs equally well (Horsting et al. 2014). American nurse-midwives in 2019 also viewed educating families about NIPT as the domain of GCs (Dettwyler et al. 2019). However, in a 2018 study, American GCs noted that the development and increased use of expanded testing would further strain resources GCs could provide (Agatisa et al. 2018). Approximately a third (30.8%, n = 37) of OBs in New Zealand in a 2017 study believed pre-test counseling should be the role of the lead maternity carer, who should be supported to do so through appropriate education and training (Filoche et al. 2017).

#### Levels of comfort providing pre-test counseling

Most research regarding comfort levels in providing pre-test counseling was conducted among obstetric professionals. Most (84%) of Australian OBs and MFMs in a 2015 study scored themselves 4 or 5 (5 being “I feel very well informed”) on 5-point likert scale about NIPT (Hui et al. 2015). In a 2016 study, the majority (83%, n = 10) of American OBs who offered expanded NIPT felt at least somewhat comfortable explaining it to others, however only 34% (n = 25) of OBs who did not order expanded NIPT felt similar levels of comfort (Mayes et al. 2016). In another 2016 study, American OBs and MFMs reported being comfortable or very comfortable discussing detection rate (n = 123, 48.2%; n = 110, 43.1%) and false positive rate (n = 125, 49.0%; n = 92, 36.1%) for common aneuploidies (Farrell et al. 2016). Significantly higher mean levels of comfort were reported by MFMs compared to OBs when discussing advantages and disadvantages of NIPT compared to other prenatal tests (Farrell et al. 2016). Obstetricians in New Zealand in 2017 reported being quite or very comfortable (40.6%, n = 52; 35.2%, n = 45) offering NIPT as a screening option for fetal aneuploidy (Filoche et al. 2017). Many also reported confidence in explaining positive predictive value, the likelihood that a fetus with a positive test result actually has the condition being tested, and fetal fraction (Filoche et al. 2017). Confidence in explaining NIPT varied significantly between OBs in a 2020 study in the Philippines, with 26.6% (n = 25) being uncomfortable explaining the test and 38.3% (n = 37) being comfortable or very comfortable (Panes K.D.P. & Javier G.C., 2020).

Genetic counselors reported confidence in explaining NIPT to pregnant people, with most (93.2%, n = 191) American GCs at least somewhat agreed that they felt confident explaining how NIPT was performed in 2014 (Horsting et al. 2014). Most (94.1%, 195) also at least somewhat agreed that they felt confident offering NIPT (Horsting et al. 2014).

#### Timing of pre-test counseling

Practice and views on the timing of offering NIPT also varied. While most (77.9%, n = 201) American MFMs and OBs reported counseling for NIPT during initial prenatal visits, (Farrell et al. 2016) some Dutch midwives reported avoiding mentioning NIPT in a person’s first visit as they felt it was not appropriate during early pregnancy as it may impact the experience of being pregnant and given the potential for miscarriage (de Vries et al. 2022). Obstetricians and midwives in New Zealand estimated 30% of women they saw raised the request for NIPT (Eastwood A. et al., 2016). In a 2014 study, 85.4% (n = 182) of UK GCs reported pregnant people had heard about NIPT prior to counseling less than 50% of the time (Horsting et al. 2014). Most Dutch healthcare providers (74%) believed that counseling for NIPT and the sampling of maternal blood should take place on different days. Midwives were more likely than OBs or sonographers to support counseling and blood sampling over separate days (Tamminga et al. 2015). Dutch midwives in a 2022 study reported mostly counseling for NIPT over two consultations: one at 7–8 weeks with a viability scan and one at 9–12 weeks with a dating scan (de Vries et al. 2022). In a study of UK prior to the clinical implementation of NIPT, OBs and midwives were less likely to indicate that the presentation and uptake of NIPT should occur on different days (74%) compared to invasive diagnostic testing (94%) (van den Heuvel et al., 2010). UK HPs in a 2016 study were less likely to believe NIPT should take place on a return visit than they were for diagnostic testing (Silcock et al. 2015).

#### Barriers to the provision of pre-test counseling

Lack of time was frequently reported as a barrier to adequate pre-test counseling (Agatisa et al. 2018; Bowman-Smart et al. 2024; Burgess et al. 2020; de Vries et al. 2022; Farrell et al. 2016; McKinn et al. 2022; Ngan et al. 2018). Midwives in the Netherlands reported time constraints requiring them to skip some parts of their explanation to provide only essential information in order to still support decision-making (de Vries et al. 2022). Healthcare professionals in Hong Kong noted time constraints were a particular issue in public sectors creating disparities in the counseling provided to women in public and private health services (Ngan et al. 2018). American GCs suggested that one clinical visit or counseling session would be insufficient to support informed decision-making about NIPT use given the growing complexity of prenatal genetics (Agatisa et al. 2018). However, in contrast to this, 45.8% (n = 55) New Zealand OBs viewed 15 min as sufficient for pre-testing counseling (Filoche et al. 2017) and Dutch midwives anticipated they would need an average of 12 min to provide counseling for NIPT if used as a first line screening test (de Vries et al. 2022). However, Dutch midwives also reported postponing counseling sessions if they felt pregnant people had not adequately prepared, such as through reading provided brochures, as there was insufficient time to explain all screening options without said preparation (de Vries et al. 2022).

The provision of pre-test counseling to people who did not speak English was also noted as challenging by GCs in the UK (Agatisa et al. 2018). Dutch midwives similarly raised language barrier as a challenge, particularly when the pregnant person brought someone with them to translate, leaving midwives uncertain about how or what information is being conveyed (de Vries et al. 2022). Other challenges noted by HPs providing pre-test counseling for NIPT included providing pre-test counseling to people with low health literacy, (Agatisa et al. 2018) complexities arising from laboratories screening for different conditions and having different approaches to consent (Perrot & Horn 2022) and concerns about the utility of expanded testing panels and whether these could be sufficiently counselled to support people to make informed choices about their pregnancies (Gammon et al. 2016).

### Post-test counseling

Experience with receiving positive NIPT results varied. In a 2014 study, 77.2% of American MFMs reported receiving positive or abnormal NIPT results (Haymon et al. 2014). In a 2016 study, approximately half (49%, n = 35) of American OBs had never received a positive result from expanded NIPT (Mayes et al. 2016).

Test results were primarily delivered in-person by European HPs, who also typically provided test users with the full NIPT report (Benachi et al. 2020). Just over half (51.3%) of American OBs believed that, if NIPT was based on sequencing fetal DNA in maternal plasma thereby providing a large amount of sequence information about the genetic parents and the fetus, all fetal sequence information should be provided to both parents. Obstetricians with more than 10 years of practice experience were more likely to support the provision of information to both parents (Benn et al. 2014). Australian HPs typically provided positive test results in-person and negative test results via a phone call (Johnston et al. 2024).

American OBs who received a positive result on expanded NIPT in a 2016 study most commonly referred to a subspecialist, such as an MFM or GC (Mayes et al. 2016). American nurse-midwives reported NIPT results were provided to pregnant people by MFMs or GCs and communicated to nurse-midwives via progress notes or the pregnant person (Dettwyler et al. 2019). Genetic counselors were viewed as the most important profession in providing post-test counseling for positive NIPT results by American OBs in 2013 (Musci et al. 2013). This contrasted with the views of New Zealand OBs in a 2017 study, who believed that high chance results should be counselled by OBs or MFMs (Filoche et al. 2017).

Most HPs reported confidence in providing post-test counseling to people who received positive NIPT results. American OBs and MFMs reported being comfortable or very comfortable discussing post-test options based on a positive NIPT result (Farrell et al. 2016). American MFMs reported significantly higher mean levels of comfort discussing post-test options following NIPT results than OBs (Farrell et al. 2016). Singaporean OBs and MFMs also reported high confidence providing post-testing counseling to people who received high chance NIPT results (Yang & Tan 2020).

Challenges experienced during post-test counseling included communicating test failures and gaining information from commercial laboratories. European HPs reported challenges in counseling for failed NIPT and suggested that pregnant people should be made aware of potential test failure in pre-test counseling (Benachi et al. 2020). Genetic counselors in America noted obtaining test results and information from commercial laboratories as a challenge, citing a lack of support interpreting no-call results and laboratory unwillingness to share proprietary data (Agatisa et al. 2018).

### Termination of pregnancy following NIPT

Most HPs in one survey believed NIPT results would significantly influence a person’s decision-making about termination of pregnancy. However, most HPs in this study viewed this influence to be reasonable (Sayres et al. 2011). Obstetricians in the United States and New Zealand raised concerns that the availability of NIPT may encourage abortion for mild or non-lethal conditions (Benn et al. 2014; Filoche et al. 2017). A small percentage of HPs in the United States and Australia believed at least one of the pregnant people in their care had ended a pregnancy based on NIPT results alone (Buchanan et al. 2014; Johnston et al. 2024). However, concerns about NIPT encouraging abortion was challenged by HPs in the Netherlands, who did not believe NIPT use would increase rates of abortion for “trivial reasons” as, for most people undergoing prenatal testing, it was a desired pregnancy (Kater-Kuipers A. et al., 2018). The perceived potential for NIPT to normalise abortion for Down syndrome was viewed as a political factor in delaying the implementation of NIPT by some HPs in the Netherlands (Van Schendel et al. 2017). Healthcare professionals in Lebanon further noted that concerns about abortion following NIPT may be culture-specific, as it was not uncommon for pregnant people in Lebanon to decline termination due to religious beliefs following a positive result (Haidar et al. 2020). However, HPs in Lebanon also questioned whether NIPT could be ethically implemented in countries, such as Lebanon, where abortion was legally prohibited (Haidar et al. 2020).

Healthcare professionals indicated they did or would offer abortion following NIPT in a small number of studies. A small percentage of American MFMs (Swaney et al. 2016) and Canadian general practitioners (Chan et al. 2018) indicated they would offer or discuss abortion following a positive NIPT result. American OBs and MFMs reported lower levels of comfort discussing the option of terminating a pregnancy following a positive result or issues related to raising a child with a serious medical condition or disability (Farrell et al. 2016). General practitioners (19.4%) in Canada were significantly more likely to offer abortion following a positive NIPT results than MFMs (0.0%), OBs (4.4%) and midwives (7.1%) (*p* = 0.009) (Chan et al. 2018). Dutch HPs expressed the view that that NIPT is not for the purposes of abortion and that it should be made clear to potential test users that NIPT is not exclusively offered to people considering an abortion (Kater-Kuipers et al., 2018).

### Education and training

Healthcare professionals predominately learned about NIPT through professional associations, (Benachi et al. 2020; Farrell et al. 2016; Gammon et al. 2016; Johnston et al. 2024) clinical or educational meetings (Benachi et al. 2020; Burgess et al. 2020; Farrell et al. 2016; Filoche et al. 2017; Hui et al. 2015; Johnston et al. 2024) and academic literature (Benachi et al. 2020; Burgess et al. 2020; Farrell et al. 2016; Filoche et al. 2017; Hui et al. 2015; Johnston et al. 2024). In Hong Kong, OBs and midwives reported colleagues and university or hospital websites to be their most common source of information (Yi et al. 2015). Differences in information sources were noted between OBs, who tended to obtain information from scientific reports, and midwives, who gained information from informal workplace training, in Hong Kong (Ngan et al. 2018).

Commercial laboratories were also a prominent source of information for American OBs and MFMs across two studies (Farrell et al. 2016; Mayes et al. 2016). Nearly half of OBs and MFMs in an Australian and New Zealand survey similarly reported direct contact with NIPT providers as means of learning about NIPT (Hui et al. 2015). Healthcare professionals in a second Australian study raised concerns about testing companies marketing NIPT directly to GPs without sufficient investment in education and training resources and drew a connection between the need for more consistent education and the commercial nature of NIPT (McKinn et al. 2022). Obstetric professionals in New Zealand raised similar concerns about the ad hoc implementation of NIPT with minimal support for healthcare providers (Filoche et al. 2017).

Healthcare providers in Australia, Europe and the United States expressed a desire for more information about NIPT for both themselves and pregnant people considering NIPT (Benachi et al. 2020; Bowman-Smart et al. 2024; Gammon et al. 2016; McKinn et al. 2022). When asked what aspects of NIPT they would like more information about, New Zealand obstetric professionals indicated “all of it” from arranging screening and providing counseling to technical aspects of the test (Filoche et al. 2017). European HPs reported difficulties staying up-to-date with information about NIPT, particularly given the rapid evolution of the technology and continuous changes in test options (Benachi et al. 2020; Gammon et al. 2016). Genetic counselors in one American study felt a professional obligation to assist in supporting the education of OBs about NIPT (Agatisa et al. 2018).

### Views on genome-wide NIPT

Healthcare professionals viewed the implementation and use of NIPT for whole genome sequencing (WGS) and whole exome sequencing (WES) as inevitable (Haidar et al. 2022; van Dijke et al. 2022). Despite this, the majority (80%) of HPs in one study opposed WGS due to lack of scientific information about and associated difficulty interpreting and managing test results, lack of clinical utility, difficulties in counseling and achieving informed consent, the future child’s autonomy and the use of NIPT for non-medical traits and the subsequent trivialisation of abortion (Haidar et al. 2022). Some HPs anticipated that WES would facilitate the implementation of diagnostic uses of NIPT given fetal DNA would also be analysed (van Dijke et al. 2022).

Healthcare professionals felt they required additional training around offering genome-wide NIPT, noting that disparities in information provided to pregnant people are likely to be exacerbated should the scope of NIPT expand (Perrot & Horn 2022). In particular, challenges with upskilling and intaking sufficient numbers of GCs (Alexander et al. 2015; Perrot & Horn 2022) and providing comprehensive pre-test counseling that sufficiently prepared NIPT users for possible results(Haidar et al. 2022; Perrot & Horn 2022) were noted.

### Views on specific uses of NIPT

#### Down syndrome (Trisomy 21)

Most HPs would or did offer NIPT for Down syndrome (Ahmed et al. 2017; Benn et al. 2014; Haymon et al. 2014; Sayres et al. 2011). While most HPs in the USA and UK indicated that Down syndrome screening should be offered to all pregnant people, (Benn et al. 2014; Silcock et al. 2015). 11% of American OBs believed it should only be offered to those who were high risk (Benn et al. 2014). Family history of Down syndrome was also an indicator for offering screening (Haymon et al. 2014). When asked whether a hypothetical, non-invasive diagnostic test should be offered if it were only available for Down syndrome, 78.2% of American OBs indicated they would offer it to all pregnant people (Benn et al. 2014).

Experiences with counseling for NIPT use for Down syndrome varied. UK HPs perceived a lower need for written consent when Down syndrome was screened for by NIPT than via invasive diagnostic testing (Silcock et al. 2015). Obstetricians and MFMs in Singapore reported high confidence discussing the clinical features of Down syndrome, the accuracy and limitations of NIPT for Trisomy 21 and options if high chance test results were returned (Yang & Tan 2020). However, correcting or balancing misinformation about Down syndrome screening was raised as a challenge. Genetic counselors in the USA believed obstetric colleagues often placed undue emphasis on NIPT as a screening test for Down syndrome during pre-test counseling, without adequately acknowledging other commonly screened conditions (Agatisa et al. 2018). Dutch midwives further reported difficulties correcting misinformation about Down syndrome that pregnant people acquired from external sources, such as media or family and friends (de Vries et al. 2022).

Some HPs raised concerns that NIPT would increase discrimination against newborns with Down syndrome (Ngan et al. 2017; Yi et al. 2015). Most HPs (60%). in Hong Kong were somewhat concerned that NIPT would result in limited autonomy for a woman to have a child with Down syndrome (Ngan et al. 2017). Healthcare professionals in another Hong Kong study were similarly concerned about pregnant people being able to choose to have a child with Down syndrome (Yi et al. 2015). Dutch HPs viewed the concern that NIPT would reduce the number of births of people with Down syndrome was not justified in their local context. Public acceptance of disability in the Netherlands and the availability of quality care for people with disabilities were seen as protective factors (Kater-Kuipers A. et al., 2018).

Healthcare professionals ranked accuracy as the most important attribute of a prenatal test for Down syndrome across discrete choice experiments in the UK, Singapore and a multi-country study (Barrett et al. 2017; Hill, Fisher, et al., 2012; Hill et al. 2016). Healthcare professionals’ preference for test accuracy diverged from the views of pregnant women, who ranked absence of miscarriage risk as most important (Barrett et al. 2017; Hill, Fisher, et al., 2012; Hill et al. 2016). Preferences for test attributes also differed between HPs from different countries, with HPs in the Netherlands, Canada and the United Kingdom placing greater emphasis on absence of miscarriage risk than HPs in other countries (Hill et al. 2016).

#### Expanded NIPT

Non-invasive prenatal testing was introduced and is widely used to screen common autosomal aneuploidies. Expanded NIPT panels provide the option to screen for a broader range of conditions, including selected rare autosomal aneuploidies, sex chromosome aneuploidies and microdeletions. Most (75%, n = 64) American OBs in a 2016 study were aware of expanded NIPT. Almost half (48%) of HPs in a 2020 European study stated that they had not used expanded NIPT (Benachi et al. 2020). Reasons for using expanded NIPT included where there was a family history of a given condition, abnormal ultrasound findings or by request by the pregnant person (Benachi et al. 2020). While most Australian HPs (53%) offered pregnant people a choice of targeted (only trisomies 21, 18, and 13 with or without sex chromosome aneuploidy) or expanded NIPT in a 2024 survey, a third did not offer or infrequently offered expanded NIPT (Johnston et al. 2024). While some American HPs in a 2016 study were optimistic about expanded NIPT for the additional information it could provide, others question its utility, noting the challenges of staying up-to-date with developing test panels and explaining expanded NIPT to pregnant people in a way that supported informed decision-making (Gammon et al. 2016). Dutch HPs suggested that if NIPT were to be made available for a broader range of conditions, it should be offered for a fixed list of disorders (Tamminga et al. 2015).

#### Sex chromosomes

##### X-linked conditions

In one American study, more than half of (58.8%) MFMs viewed it at least somewhat likely that early detection of fetal sex via NIPT would lead to better care for those at risk for X-linked conditions associated with ambiguous genitalia development (Swaney et al. 2016). Healthcare professionals in the UK viewed the availability of NIPT to determine fetal sex in pregnancies at risk of haemophilia to be beneficial as it allowed people carrying a female foetus to avoid invasive testing (Hill et al. 2012). While HPs acknowledged positive developments in treatment for haemophilia and subsequent decreases in requests for diagnosis with a view to abortion, some felt it was inappropriate or unethical to terminate pregnancies for clinically mild or moderate haemophilia (Hill et al. 2012). Some services had policies in place to refer to specialist services where people with a family history of mild or moderate haemophilia considering termination to avoid possible bias or pressure from haemophilia specialists (Hill et al. 2012). Some HPs noted that NIPT was initially offered to all haemophilia carriers at their service, but this later changed to only offer NIPT with invasive testing either because of budget restrictions or an evolving belief that NIPT should only be used if it were to change the management of the pregnancy (Hill et al. 2012).

#### Sex chromosome aneuploidies (SCAs)

Most HPs reported that they offered NIPT for SCAs or that it was available through their place of work (Stevens et al. 2023; Yang & Tan 2020). Reasons for offering SCA screening included the desire to support autonomy and the absence of additional cost (Yang & Tan 2020). Canadian HPs suggested testing for Klinefelter syndrome was unnecessary, given the lack of medical indication for abortion, and that testing for Turner syndrome should only be performed following ultrasound indication of severe medical complications for which abortion could be considered (Haidar et al. 2022).

Most (94.1%) American GCs had experience with positive NIPT results for SCAs (Fleddermann et al. 2019). Experience with positive NIPT results for Turner syndrome (45,X; 89.8%) were most commonly reported, followed by Klinefelter syndrome (47,XXY; 60.9%), Triple X syndrome (47,XXX; 52.3%), Jacobs syndrome (47,XYY; 31.13%) and other SCA results (7.8%) (Fleddermann et al. 2019). Incidental diagnosis of a SCA via NIPT was raised as an ethical concern by obstetric sonologists in Australia and New Zealand (Hui et al. 2015). Genetic counselors working in paediatric practice agreed that a karyotype should be offered to all newborns following a positive NIPT for SCA regardless of the presence of dysmorphic features (Fleddermann et al. 2019). The frequency of offering maternal karyotype following NIPT results was highly variable, most commonly offered following positive NIPT results for Turner or Triple X syndromes where fetal diagnostic testing was normal or declined (Fleddermann et al. 2019).

While the need for guidelines for counseling for SCAs was highlighted by HPs in Australia,(McKinn et al. 2022). HPs in the United States and Singapore generally reported high confidence in discussing SCAs (Fleddermann et al. 2019; Yang & Tan 2020). In one study, American GCs’ confidence was significantly associated with length of practice experience, with those who had been practising for less than 5 years reporting lower confidence than counselors who had been practising for 5 years or longer for every condition except for Turner syndrome (Fleddermann et al. 2019). Despite high reported confidence, pre-test counseling practice among HPs varied. Just over half (59.2%) of American obstetric care providers reported often or always discussing SCAs in pre-test counseling (Farrell et al. 2016).

#### Non-medical fetal sex determination

Healthcare professionals viewed the use of NIPT for non-medical fetal sex determination as an ethical issue, (Hui et al. 2015) primarily arising from the use of NIPT for sex selective abortion (Alexander et al. 2015; Claesen-Bengtson et al. 2024; Haidar et al. 2020; McKinn et al. 2022; Swaney et al. 2016). Sex selective abortion was viewed by HPs as an inappropriate use or misuse of NIPT (Haidar et al. 2020; Swaney et al. 2016). Healthcare professionals noted that sex selective abortion could be motivated by cultural norms that valued male children (Claesen-Bengtson et al. 2024; Haidar et al. 2020) as well as for purposes of family balancing (Claesen-Bengtson et al. 2024; Haidar et al. 2020, 2022). While no HPs in an Australian study had encountered sex selection in practice, (McKinn et al. 2022) a minority of HPs in the United Kingdom believed some pregnant people in their care had requested testing for fetal sex for social reasons (Alexander et al. 2015).

Rates of discussing and offering NIPT for fetal sex determination varied. Despite concern, some HPs felt obligated to discuss and offer NIPT for fetal sex out of respect for the autonomy (Agatisa et al. 2018; Claesen-Bengtson et al. 2024). In one study, 71% of American and Canadian GCs reported disclosing the ability of NIPT to determine fetal sex during their initial pre-test counseling (Stevens C. et al., 2023). In contrast, studies with Pakistani OBs (Ahmed et al. 2017) and American obstetric professionals (Sayres et al. 2011) reported only 31% and 21% would offer NIPT for non-medical fetal sex determination respectively. A minority of HPs reported only discussing the capability of NIPT to determine fetal sex upon request (6.3%) or that they directed pregnant people to alternative methods of obtaining information about fetal sex (29.1%), such as direct-to-consumer prenatal screening tests or early ultrasound. However, some Belgian GCs reported adopting a directive approach to counselling in the context of fetal sex, placing moral weight on certain actions and reasons. Belgian GCs also referenced their right to conscientiously object to sex-selective abortion, reporting withholding or delaying information or referring couples to other providers (Claesen-Bengtson et al. 2024).

Misinformation among pregnant people was identified as a challenge for the provision of pre-test counseling. Genetic counselors in the USA and Canada reported challenges arising from increasing numbers of pregnant people who believed the primary function of NIPT was to provide information about fetal sex (Agatisa et al. 2018; Stevens et al. 2023). Genetic counselors noted challenges in providing genetic counseling for NIPT where other healthcare providers had described NIPT as a test for gender, particularly during post-test counseling following the return of positive results (Stevens et al. 2023). Healthcare professionals additionally noted challenges providing counselling where pregnant people conflated terminology relating to sex and gender. While most HPs reported that people commonly conflated sex and gender, GCs rarely reported discussing differences during their clinical practice (Stevens et al. 2023). A minority of GCs in the USA and Canada reported pregnant people who were interested in NIPT for fetal sex subsequently declining the test once informed about its function for screening fetal aneuploidy (Stevens et al. 2023).

Possible benefits of fetal sex determination were noted by a minority of HPs. These were the belief that early parental knowledge of biological sex was thought to facilitate bonding (McKinn et al. 2022) and that sex selection could be viewed positively in specific situations within a cultural context (Alexander et al. 2015).

#### Microdeletions and microduplications

Healthcare professionals’ views on the use of NIPT for the detection of microdeletions and microduplications were varied (Gammon et al. 2016; Haidar et al. 2022). In one study, 34.1% of Canadian HPs opposed this use of NIPT, while 8.9% were in favour and 57.0% were undecided (Haidar et al. 2022). Despite this, studies in Europe, the USA, Canada and Singapore reported that most HPs offered NIPT for microdeletions and microduplications (Benachi et al. 2020; Stevens et al. 2023; Yang & Tan 2020).

Reasons for supporting NIPT for microdeletion and microduplication detection included supporting autonomy, (Yang & Tan 2020) that this use of the test was not associated with additional cost (Yang & Tan 2020) and the opportunity to gain more information about the fetus (Gammon et al. 2016). Apprehensiveness arose from uncertainty around the utility of detecting microdeletions and microduplications, (Gammon et al. 2016) low test sensitivity,(Burgess et al. 2020) challenges keeping up-to-date with ever-changing test panels (Gammon et al. 2016) lack of access to validation data (Agatisa et al. 2018) as well as the complexities explaining them to pregnant people in order to support informed decision-making (Agatisa et al. 2018; Gammon et al. 2016).

#### Monogenic conditions

Willingness to offer NIPT for monogenic conditions varied significantly. A survey of American MFMs reported 89.4% of respondents were willing to offer NIPT for monogenic conditions in 2014 prior to its availability, (Haymon et al. 2014) whereas 38.0% of American and Canadian GCs reported offering single gene NIPT in 2023 (Stevens C. et al., 2023). Healthcare professionals in the UK viewed NIPT for monogenic conditions positively, but felt it should be offered through specialist services, such as genetic counseling or fetal medicine (Hill et al. 2013). Where NIPT for monogenic conditions was globally available but yet to be locally implemented, such as in the Netherlands, HPs viewed its introduction as a matter of time (van Dijke et al. 2022). Some Dutch HPs noted the potential for NIPT to be used both as a diagnostic and screening test where used to diagnose monogenic conditions and screen for chromosomal anomalies (van Dijke et al. 2022).

More than half (55%) of obstetric HPs indicated they would offer NIPT for congenital adrenal hyperplasia and 82% would offer NIPT for cystic fibrosis in 2011 study (Sayres et al. 2011). Canadian health professionals in a 2022 study viewed NIPT for cystic fibrosis as holding great potential with the possibility to replace invasive diagnostic procedures should it become clinically available (Haidar et al. 2022). They suggested that NIPT for cystic fibrosis should be offered only to couples known to be carriers, rather than be universally available (Haidar et al. 2022). Clinical geneticists and GCs in a discrete choice experiment in the UK placed importance on test accuracy and early testing as characteristics of prenatal tests for cystic fibrosis, compared to adult patients and carriers of cystic fibrosis who placed importance on test safety (Hill et al. 2014).

Healthcare professionals in the United Kingdom perceived an existing pressure for pregnant people to test prenatally for sickle cell disorders, with HPs viewed as the primary source of this pressure (Hill et al. 2017). Healthcare professionals believed the development of NIPT to diagnose sickle cell disorder would increase pressure to test (Hill et al. 2017).

#### Polygenic conditions

In a 2022 Canadian study, 92.8% of HPs opposed the use of NIPT to test for physical and behavioural attributes such as eye colour, intelligence and sexual orientation (Haidar et al. 2022). More than half (52.7%, n = 17) were also opposed to screening for mental disorders such as schizophrenia or bipolar disorder (Haidar et al. 2022). In a 2011 study, approximately half of nurses and OBs reported they would find it acceptable to offer prenatal genetic testing for autism (56%) or schizophrenia (45%) (Sayres et al. 2011).

#### Childhood-onset conditions

Healthcare professionals were more favourable towards using NIPT for childhood-onset conditions, such as autism and leukemia, than late or adult-onset conditions (Haidar et al. 2022; Yotsumoto et al. 2012). Most Canadian HPs were in favour (19.8%) or somewhat in favour (22%) of screening for childhood-onset conditions, while 30.2% were opposed (Haidar et al. 2022).

#### Late or adult-onset conditions

Healthcare professionals varied in their views on the acceptability of NIPT for late or adult onset conditions (Yotsumoto et al. 2012). A Canadian study found that 58.6% of HPs were opposed to offering NIPT for late onset conditions, while 12.7% were somewhat in favour and 9.4% were in favour (Haidar et al. 2022). Clinical geneticists, GCs and general practitioners in this study were significantly (p < 0.001) less likely to be in favour of testing for predispositions to late onset conditions than nurses, midwives or OB gynecologists (Haidar et al. 2022). Another study reported that 21% of obstetric professionals would offer testing for increased risk for adult-onset conditions (Sayres et al. 2011).

Reasons against offering NIPT for late-onset conditions included that NIPT results often represent a risk factor rather than diagnosis (Haidar et al. 2022) and that they may provide false reassurance, particularly when screening for multifactorial conditions such as cancer (Bennett et al. 2016). In the context of BRCA testing, HPs raised concerns that pregnant people may be pressured to test and terminate and noted the dominant inheritance pattern of BRCA could result in multiple terminations for some people (Bennett et al. 2016). While some HPs in the UK predicted difficulties condoning termination for BRCA-affected pregnancies, others suggested testing should only be available where abortion was being considered (Bennett et al. 2016). Healthcare professionals additionally proposed reasons against offering NIPT related to the future child. These include the potential future development of a treatment for the detected condition, labelling of the future child and lack of immediate impact on the child’s health and development (Haidar et al. 2022). Even if a late onset condition were diagnosable, some Canadian HPs argued that the future child would “have enough time to live and lead a normal life” prior to condition onset (Haidar et al. 2022).

Support for the use of NIPT for late-onset conditions was given in the context of extensive pre-test counseling and consultation with a psychologist (Bennett et al. 2016; Haidar et al. 2022). In the context of BRCA testing, some UK HPs viewed the development of NIPT as an advancement in reproductive autonomy through providing pregnant people and their partners with more reproductive options (Bennett et al. 2016). Canadian HPs were cautious about framing the conditions for which they support the use of NIPT, with some focussing on test reliability (Haidar et al. 2022). Some HPs supported testing for late-onset conditions, noting that it is difficult for a healthcare professional to determine whether a condition is sufficiently severe to justify testing and that a patient’s lived experience of a condition may influence desire to test and should be taken into account (Haidar et al. 2022). For example, HPs predicted BRCA NIPT may be beneficial for patients who had psychosocial trauma from their experience with cancer: providing reassurance, preventing feelings of guilt associated with passing on the mutation or providing more time to process the results if continuing the pregnancy (Bennett et al. 2016).

#### RhD blood typing

Rhesus D blood typing was viewed as an appropriate reason to offer and use NIPT (Ahmed et al. 2017; Haymon et al. 2014; Sayres et al. 2011; Yotsumoto et al. 2012). Following the implementation of fetal RhD genotyping in the UK, most HPs reported that extra time was required to record and manage results. Training and education for staff was reported to take approximately 30 min. No additional costs or resources associated with the transport and management of samples were reported (Ryczek E. et al., 2020).

#### Maternal neoplasm

One study explored the views and experiences of GCs in the USA with maternal neoplasm (Giles et al. 2017). While awareness was not associated with change in pre-test counseling, GCs who personally experienced NIPT detecting maternal neoplasm were significantly more likely to discuss this possible outcome in subsequent pre-test counseling (Giles et al. 2017). Of those who discussed maternal neoplasm as a possible outcome, some explicitly acknowledged the potential detection of maternal cancer whereas others alluded to maternal health factors, discussed it on a case-by-case basis or discontinued offering NIPT. Approximately half (52%) felt uncomfortable counseling for maternal neoplasm and most (91%) wanted additional data and guidelines to support their counseling practice. All, except one, participants stated they would disclose maternal neoplasm findings to NIPT users if it were clearly documented in the test report. A quarter (25%) of GCs were unsure about what recommendations or referrals they would make following detection of maternal neoplasm (Giles et al. 2017).

#### Secondary test use

Half (50.3%) of American OBs indicated that the consent of both parents should be required for any use of sequence information beyond initial fetal screening and diagnosis, such as to test newly discovered genetic associations or paternity testing (Benn et al. 2014). While 19.2% of Canadian HPs in one survey were in favour of using NIPT for paternity testing, 39.0% survey respondents were opposed (Haidar et al. 2022). Canadian HPs who were interviewed following this survey unanimously opposed paternity testing in the absence of a medical reason to do so (Haidar et al. 2022).

### Perceived benefits to pregnant people

Healthcare professionals viewed the availability of NIPT early in pregnancy as a benefit to pregnant people (Gammon et al. 2016; Hill et al. 2013, 2017; McKinn et al. 2022; Ngan et al. 2017). Early testing was perceived to be reassuring for parents when a negative result was returned (Agatisa et al. 2018; Bennett et al. 2016; Hill et al. 2013; McKinn et al. 2022; Moller et al. 2018; Orzechowski et al. 2021). Reassurance was understood as arising from both being informed that the baby was unlikely to have a condition(Moller et al. 2018) as well as the knowledge that they had done all they could non-invasively to ascertain the health of their child (Agatisa et al. 2018).

Supporting informed decision-making was also perceived as a key benefit of NIPT (Hill et al. 2017; McKinn et al. 2022). The early availability of NIPT was viewed positively as it allowed for greater time for deliberation (Bennett et al. 2016) and to make decisions about their pregnancy more aligned with their personal values and preferences (Agatisa et al. 2018). While informed decision-making about whether to continue or terminate a pregnancy was recognised by some HPs as the primary aim of prenatal screening, (Kater-Kuipers A. et al., 2018) others noted additional benefits for those who were testing for information only (Agatisa et al. 2018) or to prepare for the birth of a child with a disability or condition (Kater-Kuipers A. et al., 2018; McKinn et al. 2022). Genetic counselors practising in the USA noted how NIPT could be particularly beneficial to pregnant people living in conservative areas, where local cultural or healthcare services have the potential to influence decision-making and access to reproductive healthcare. Access to NIPT in conservative areas was considered to aid pregnant people to access information about their pregnancy in a way that would be unlikely to alert family or community members about the decision to undertake screening and potential associations between screening and beliefs on disability or abortion (Agatisa et al. 2018).

Many HPs viewed NIPT as beneficial for people who wished to avoid diagnostic testing (Horsting et al. 2014). The absence of miscarriage risk was the perceived benefit of avoiding diagnostic testing by most HPs (Agatisa et al. 2018; Alexander et al. 2015; Bennett et al. 2016; Gammon et al. 2016; Hill et al. 2013, 2017; Horsting et al. 2014; McKinn et al. 2022; van Dijke et al. 2022). Non-invasive prenatal testing was perceived to support pregnant people to focus on what information was valuable to them without having to weigh this against the risk of miscarriage (Bennett et al. 2016). Notably, GCs in one UK study reported that pregnant people appeared not only to value the absence of miscarriage, but also the ability to avoid the entire invasive procedure itself (Alexander et al. 2015). UK HPs also noted that the simplicity of the blood draw for NIPT made it more accessible for pregnant people to have locally without needing to travel to a specialist centre (Hill et al. 2013).

### Perceived challenges for pregnant people

Healthcare professionals raised concerns about the amount and complexity of information to be conveyed during counseling for NIPT and the impact of this on pregnant people. Anxiety, tension, distress and uncertainty were identified as possible outcomes of inadequate pre-test counseling (de Vries et al. 2022; McKinn et al. 2022). Possible anxiety was also noted where NIPT was used as a second-line screening test, thus delaying definitive diagnoses,(Filoche et al. 2017) and where low positive predictive values led to unnecessary diagnostic testing or abortion (McKinn et al. 2022). Healthcare professionals additionally expressed concern about misunderstanding among pregnant people, particularly arising from test results that could be difficult to interpret(de Vries et al. 2022) and that test failures or inconclusive results that could cause distress and delayed diagnosis (Alexander et al. 2015; Hill et al. 2013; Hui et al. 2015). Potential misunderstanding of NIPT to be a replacement for ultrasound or other diagnostic testing resulting in false reassurance and delayed identification of abnormalities was also raised (Hui et al. 2015; McKinn et al. 2022).

Healthcare professionals in Japan raised concerns that insufficient pre-testing counseling may lead to increased test participation without full consideration of possible outcomes (Yotsumoto et al. 2012). Obstetricians, nurses and midwives in Hong Kong were similarly concerned about uptake of NIPT without adequate information (Ngan et al. 2017). Healthcare professionals in the UK raised concerns about pregnant people opting into NIPT without prior consideration of possible results (Hill et al. 2013). Dutch HPs expressed concerns that NIPT users may be faced with unwanted choices about whether to continue pregnancies if they are not adequately informed during pre-test counseling (Kater-Kuipers et al., 2018).

While some HPs raised concerns around the routinisation of NIPT and subsequent pressure to test (Ahmed et al. 2017; Alexander et al. 2015; Haidar et al. 2020; Hill et al. 2017; Sayres et al. 2011) and terminate,(Ahmed et al. 2017; Hill et al. 2013, 2017) others indicated being largely unconcerned (Birko et al. 2019; Filoche et al. 2017). Some Dutch midwives noted the burden of choice arising from test availability, acknowledging some people would prefer not to be aware of their prenatal screening options. Midwives in this study attributed this preference as a means of the pregnant person protecting their child (de Vries et al. 2022). Genetic counselors in the USA and HPs in the Netherlands raised concerns about the perceived ease of NIPT, suggesting that invasiveness and miscarriage risk acted as safeguards in diagnostic testing that encouraged pregnant people and providers to carefully consider whether to proceed with testing and possible outcomes (Alexander et al. 2015; Kater-Kuipers A. et al., 2018).

### Impact on people with disabilities

Concerns about the impact of NIPT use for people with disabilities were raised by Canadian and Lebanese HPs in one study. Healthcare professionals worried that routine use of NIPT could increase abortion rates thus decreasing the number of people with disability in the population and potentially resulting in increased discrimination and stigmatisation (Haidar et al. 2020). In particular, concerns were raised about negative social reactions and potential opposition from disability rights organisations to public NIPT screening programs on the basis of the expressivist objection (Haidar et al. 2020).

### Cost and insurance coverage

Cost was identified as a significant barrier to NIPT access (Benachi et al. 2020; Birko et al. 2019; Brewer et al. 2017; Eastwood A. et al., 2016; Filoche et al. 2017; Hill et al. 2017; Hui et al. 2015; Johnston et al. 2024; Kater-Kuipers A. et al., 2018; Orzechowski et al. 2021; Zhai et al. 2016). The prohibitive cost of NIPT was identified as an issue of justice and equity (Filoche et al. 2017; Haidar et al. 2020; McKinn et al. 2022; Orzechowski et al. 2021; Yi et al. 2015). Due to cost, some MFMs in the USA and OBs in the Philippines opted not to offer NIPT at all (Haymon et al. 2014; Panes K.D.P. & Javier G.C., 2020). Some HPs also indicated that cost influenced whether they offered NIPT to a given individual (Benachi et al. 2020; Birko et al. 2019; Filoche et al. 2017; Mayes et al. 2016; Minear et al. 2015). Despite this, HPs in the UK and Pakistan often ranked cost as the least important aspect of NIPT when compared to other characteristics, such as test accuracy or availability early in pregnancy (Ahmed et al. 2017; Hill et al. 2017). Healthcare professionals in one Australian study raised concerns that pregnant people were opting for invasive diagnostic testing, with its associated miscarriage risk, following intermediate chance results from CFTS due to the prohibitive cost of NIPT (McKinn et al. 2022).

Healthcare professionals had diverse views about public funding for NIPT. Many HPs in Australia, New Zealand and Canada believed NIPT should be at least partly funded through the public healthcare system, (Birko et al. 2019; Filoche et al. 2017; McKinn et al. 2022) with some Canadian HPs supporting publicly funded access to NIPT for all women (Birko et al. 2019). Most HPs supported funding for NIPT as a first-line screening testing (Filoche et al. 2017) and some supported funding for NIPT as a second line screen (Filoche et al. 2017; Yi et al. 2015). All OBs in one New Zealand study supported public funding for Trisomy 21 detection through NIPT, with high levels of support for public funding for other common trisomies. There was some support for publicly funded NIPT for sex chromosome aneuploidies, fetal sex, Huntington’s disease and muscular dystrophy in the same study (Filoche et al. 2017). Australian HPs viewed public funding for NIPT as particularly beneficial for those living in regional or rural areas with limited access to diagnostic testing (McKinn et al. 2022). Concerns around public funding for NIPT included the potential loss of HPs’ skills around diagnostic testing (Filoche et al. 2017) and the financial burden on the public healthcare system if NIPT was offered to all pregnant people (Gammon et al. 2016; Haidar et al. 2020). However, some HPs viewed NIPT as a cheaper screening option compared to other prenatal screening tests in terms of cost to the public healthcare system as it reduced the need for subsequent diagnostic testing (Alexander et al. 2015; Gammon et al. 2016).

Insurance coverage was also viewed as a barrier to accessing NIPT by OBs and GCs in the USA (Brewer et al. 2017; Horsting et al. 2014). Insurance coverage influenced whether HPs in the USA offered NIPT(Agatisa et al. 2018; Haymon et al. 2014; Horsting et al. 2014; Mayes et al. 2016) as well as which brand of NIPT was used (Agatisa et al. 2018) Most (81.5%) OBs in one American study reported they would offer NIPT as a first-line screening test for all pregnant people if covered by insurance (Brewer et al. 2017). Approximately two thirds of Chinese perinatologists and OBs (70.4%, n = 276) and HPs in another study (61%) felt insurance companies had an obligation to pay for the cost of prenatal tests (Sayres et al. 2011; Zhai et al. 2016). However, some Canadian and Lebanese HPs believed insurance companies would be unlikely to fund NIPT for all pregnant people given its cost relative to other prenatal tests (Haidar et al. 2020).

### Views on regulation of NIPT

Although most HPs agreed that NIPT should be regulated, there were diverse views about who should be the regulating body. Most (83.4%) American OBs supported the regulation of NIPT use through professional guidelines (Benn et al. 2014). There was varied support for government regulation of NIPT, with 43.7% of American OBs supporting federal regulation (Benn et al. 2014) compared to 7% of HPs in Hong Kong (Yi et al. 2015). Chinese OBs and perinatologists supported the establishment of clear indicators for NIPT use by governmental health departments (Zhai et al. 2016). Healthcare professionals in Australia and New Zealand were generally supportive of government involvement in the management of NIPT results (Filoche et al. 2017; Hui et al. 2015). There were low levels of support for direct-to-consumer testing, (Benn et al. 2014; Sayres et al. 2011) with 47.2% of American OBs supporting a ban (Benn et al. 2014).

Healthcare professionals in America and Germany raised concerns that direct-to-consumer testing would or did leave consumers with inadequately information and support (Gammon et al. 2016; Orzechowski et al. 2021). German OBs additionally raised concerns about false marketing claims in direct-to-consumer testing (Orzechowski et al. 2021). Lebanese HPs noted the influential nature of pharmaceutical companies to market physicians to prescribe drugs in their local context and worried about the potential influence of financial profit on HPs’ provision of NIPT (Haidar et al. 2020).

Healthcare professionals were generally supportive of government management of results, with 91.5% of New Zealand OBs supporting government management of health information gained from NIPT(Filoche et al. 2017) and 84% of Australian OB and MFMs in favour of a national voluntary register linking data and written consent for follow-up of pregnancy outcome (Hui et al. 2015). Two thirds (67.9%) of American OBs supported the provision of de-identified data to a government public health agency to track the incidence of detected genetic conditions (Benn et al. 2014).

## Discussion

To our knowledge, this is the first systematic review that provides comprehensive insight into the views and experiences of HPs with non-invasive prenatal testing globally. Healthcare professionals generally viewed NIPT as a positive development for supporting the autonomous and informed decision-making of pregnant people and their partners through providing information about their pregnancy. However, HPs also reported several challenges in providing sufficient support during pre-test counseling and questioned the utility of some uses of the test.

The aim of providing NIPT to support reproductive autonomy is operationalised through informed decision-making (Kater-Kuipers et al. 2020). Informed decision-making is supported through the provision of pre-test counseling, which should provide the pregnant person to be informed about the characteristic, risks and benefits and implications of possible test outcomes and enabled to make a voluntary decision about whether to use screening (Kater-Kuipers et al. 2020). However, HPs in this review raised concerns about supporting adequately informed decision-making for NIPT, particularly in context of expanded NIPT (Alexander et al. 2015; Filoche et al. 2017; Haidar et al. 2022; Perrot & Horn 2022; Tamminga et al. 2015). Reported barriers to the provision of adequate pre-test counseling included inadequate physician knowledge, (Agatisa et al. 2018; Burgess et al. 2020; Haidar et al. 2020; Yi et al. 2015) time constraints (Agatisa et al. 2018; Burgess et al. 2020; de Vries et al. 2022; Farrell et al. 2016; McKinn et al. 2022; Ngan et al. 2018; van der Steen et al. 2019) and the availability of direct-to-consumer testing (Gammon et al. 2016; Orzechowski et al. 2021). Some HPs additionally questioned the feasibility of providing non-directive pre-test counseling (van der Steen et al. 2019). While there is no universally agreed definition, non-directive counseling typically emphasises comprehensive and value-neutral information provision (Hodgson & Spriggs 2005; Jamal et al. 2020; Warton et al. 2023). Non-directive approaches to prenatal genetic counseling have been critiqued for the feasibility of neutral counseling, (Rentmeester 2001; Weil 2003; Welkenhuysen et al. 2001) inadequately acknowledging the social context within which prenatal testing decisions are made (Mackenzie & Stoljar 2000) and whether it adequately facilitates understanding (Dive & Newson 2018; Grant & Flint 2007). Reproductive deliberation has been suggested as an alternative that recognises the important role of genetics professionals in the decision-making process, allowing them to be responsive to requests by pregnant people for value judgements, while maintaining the ability of pregnant people to lead the counseling process and their decisional responsibility (Warton et al. 2023). Moreover, this review indicates that pre-test counseling is increasingly provided by HPs without specialist genetics training, raising questions around whether these professions are being assisted to access education about how to approach pre-test counseling in order to best support informed decision-making.

While almost all HPs supported the provision of NIPT to people with high risk pregnancies, (Alexander et al. 2015; Benachi et al. 2020; Buchanan et al. 2014) support for universal access varied. Support for universal access appeared to increase the longer NIPT had been available in clinical practice,(Brewer et al. 2017; Horsting et al. 2014; Martin et al. 2018; Minear et al. 2015; Suskin et al. 2016; Yotsumoto et al. 2012) however this support did not appear to be an indicator of whether providers offered NIPT to all pregnant people (Buchanan et al. 2014; Haymon et al. 2014; Horsting et al. 2014; Suskin et al. 2016). Current clinical guidelines recommend NIPT be offered to all singleton pregnancies for the screening of common autosomal trisomies and sex chromosome aneuploidies (Dungan et al. 2023; Royal Australian and New Zealand College of Obstetricians and Gynaecologists, 2024). While evidence for the use of expanded NIPT for rare chromosomal trisomies and copy number variants is generally considered to be insufficient in current clinical guidelines, (Dungan et al. 2023; Royal Australian and New Zealand College of Obstetricians and Gynaecologists, 2024) the American College of Medical Genetics and Genomics (ACMG) suggests NIPT be offered for 22q11.2 deletion syndrome (Dungan et al. 2023). The positive predictive value of 22q11.2 has been found to range from 18.0% to 98.7% in high risk pregnancy cohorts (Hammer et al. 2024). Low positive predictive values present challenges for prenatal counselling and may lead to terminations of unaffected pregnancies. Knowledge of test validation and performance were influential factors in whether HPs in this review viewed it appropriate to offer universal access to NIPT (Suskin et al. 2016). Recent research suggests NIPT to be a reliable prenatal test for common autosomal trisomies (Jayashankar et al. 2023; Lee et al. 2019). NIPT was demonstrated to have high sensitivity (> 90%) and specificity (> 98%) when used for common autosomal trisomies in a recent review (Jayashankar et al. 2023). Greater variations were reported among positive predictive values of NIPT when used for Trisomy 21 (78.6%%–100%), Trisomy 18 (58.7–100%) and Trisomy 13 (33.3%–100%) (Jayashankar et al. 2023). However, test performance varies significantly, particularly for expanded uses of NIPT (Kim et al. 2024; Xue et al. 2022). Variations in support for universal access to NIPT may reflect challenges for HPs in remaining up to date with the clinical guidelines and test performance for a rapidly developing technology. Test performance for NIPT among all risk populations is greatest for the detection of Down syndrome, which may explain some HPs’ framing of NIPT as a Down syndrome test.

Comparatively little literature reported the experiences of HPs providing post-test counseling. Most HPs indicated that counseling for high chance NIPT results was managed by OBs, MFMs or GCs (Filoche et al. 2017; Mayes et al. 2016; Musci et al. 2013). While high confidence was reported among HPs who provided post-test counseling, (Farrell et al. 2016; Yang & Tan 2020) communicating test failures was highlighted as a particular challenge (Benachi et al. 2020). “Failed” or “no call” NIPT may be caused by low fetal fraction, maternal factors and technical issues (Kong et al. 2024; Yaron 2016). While existing literature and some HPs in this review suggest the possibility of test failures should be discussed during pre-test counseling, (Benachi et al. 2020; Murphy et al. 2020; Yaron 2016) no HPs included in this review explicitly reported discussing test failure during pre-test counseling. Test failures, particularly in the absence of comprehensive pre-test counseling, may result in distress among pregnant people (Lewis et al., 2016; Murphy et al. 2020). There is no consensus about how to manage NIPT failures (Zhang et al. 2021). The American College of Obstetricians and Gynecologists (ACOG) recommends failed NIPT tests be followed with genetic counseling, ultrasound evaluation and diagnostic testing (American College of Obstetricians and Gynecologists 2024). By contrast, the International Society for Prenatal Diagnosis (ISPD) suggests laboratories and clinicians establish management pathways which may include ultrasound, repeat NIPT, diagnostic testing or other screening tests such as CFTS (Hui et al. 2023). Some research suggests the cause of test failure should be determined and used to guide HPs on next steps (Zhang et al. 2021) whereas others propose initially choosing providers with lowest technical failure rates as a possible strategy (Yaron 2016). Test failures may thus pose a dilemma for HPs and pregnant people in deciding next steps, (Yaron 2016) indicating a need for additional provider education to support reproductive decision-making.

Healthcare professionals’ views on the use of NIPT for various conditions differed on the basis of test performance and perceived clinical utility. Healthcare professionals generally agreed about the offering of NIPT for common autosomal trisomies. This aligns with research reporting reliable test performance of NIPT for these conditions (Jayashankar et al. 2023; Lee et al. 2019) and the severe or life-threatening symptoms associated with Trisomies 18 and 13. However, HPs in this review did emphasise challenges in counseling for Down Syndrome screening given misunderstanding among pregnant people about NIPT or the condition itself (Agatisa et al. 2018; de Vries et al. 2022). Views about expanded uses of NIPT varied significantly. For example, while most HPs offered NIPT for SCAs,(Stevens C. et al., 2023; Yang & Tan 2020) some questioned the utility of the test in the absence of medical indication for abortion (Haidar et al. 2022; Yang & Tan 2020). Healthcare professionals’ uncertainty about the clinical utility of screening for SCAs is reflected in clinical guidelines and academic discourse, where positions range from suggesting prospective parents be given the option to accept or decline sex chromosome analysis to arguments against the clinical implementation of NIPT on the basis of test validity (Johnston et al. 2023). Concerns were also raised about the potential for sex-selective termination, (Alexander et al. 2015; Haidar et al. 2020; McKinn et al. 2022; Swaney et al. 2016) which HPs viewed as a misuse of NIPT (Haidar et al. 2020; Swaney et al. 2016). While there are some reports of NIPT is use for sex-selective termination, (Ravitsky et al. 2021) a review of global practice concluded more rigorous evidence was required to determine whether and how often sex selective termination occurred following NIPT (Bowman-Smart et al. 2020). The clinical utility of NIPT for microdeletions and duplications was also queried (Agatisa et al. 2018; Burgess et al. 2020; Gammon et al. 2016). Clinical guidelines vary somewhat in their recommendations regarding NIPT screening for microdeletions and microduplications. While the ISPD and the ACMG generally do not recommend NIPT screening for microdeletions and microduplications due to insufficient evidence, (Dungan et al. 2023; Hui et al. 2023). ACMG suggests that NIPT for 22q11.2 deletions be offered to all people accessing prenatal care (Dungan et al. 2023).

While healthcare professional’s views on the use of NIPT for adult-onset conditions varied, screening for adult-onset conditions was generally seen as less favourable than screening for conditions with onset in childhood (Haidar et al. 2022; Yotsumoto et al. 2012). Reasons provided against screening for adult-onset conditions included that NIPT was not diagnostic as well as concerns about the impact of a positive result on the future child (Haidar et al. 2022). Bioethics discourse provides no consensus regarding the ethical acceptability of screening for adult-onset conditions. Some argue that NIPT should not be offered for adult-onset conditions in instances where termination would not be considered (Deans et al. 2015; Duncan et al. 2006; MacLeod et al. 2013). Some ethicists argue that moral reasoning around screening for adult-onset conditions should apply equally to both fetuses, where tested for information-only, and children given both are on the same trajectory to adulthood. Reasons provided against screening for adult-onset conditions in such instances include that the test would violate the future child’s privacy and breach confidentiality on the part of the healthcare professional (Deans et al. 2015). However, others argue that pregnant people should have access to NIPT for adult-onset conditions with sufficient pre-test counseling irrespective of their intention to terminate (Bowman-Smart & Taylor-Sands 2021; Marks et al. 2023). Proponents of this position suggest that there is insufficient evidence to conclude screening for adult-onset conditions would significantly harm the future child (Bowman-Smart & Taylor-Sands 2021; Marks et al. 2023) and that comparing the moral interests of a fetus with an existing child draws a false equivalence (Bowman-Smart & Taylor-Sands 2021). One study employed a survey of people and partners who had used NIPT to argue that restricting access to NIPT for adult-onset conditions would lack public support. The authors of this study also advocate for access to NIPT of adult-onset conditions with a caveat that is must be accompanied by sufficient pre-test counseling (Marks et al. 2023). Participants in this survey were not provided with information about the nature of adult-onset conditions or possible implications of prenatal screening. Precisely because of this need for sufficient counseling to achieve informed decision-making, as acknowledged by the authors, it is unclear that the survey results can be interpreted as indicative of informed public opinion regarding the screening of adult-onset conditions. Further research is required to understand the views of HPs and prospective parents to inform considerations of the acceptability of NIPT for adult-onset conditions.

### Limitations

The methodology of this review was designed to capture all relevant research on healthcare professionals’ views on and experiences with non-invasive prenatal testing to date. While dates have been emphasised throughout this review where comparisons are made, the rapid implementation and development of NIPT means that earlier studies may not reflect current perspectives or clinical context. Further research may thus be required to investigate the views and experiences of healthcare professionals in respect to the current landscape where only earlier literature exists.

## Conclusion

Healthcare professionals play a critical role in facilitating the access to and decisions by pregnant people around prenatal genetic testing. This review systematically examined primary empirical research on HPs’ views on and experiences with NIPT globally. Healthcare professionals' views on who NIPT should be offered to, and which conditions should be screened for, were largely influenced by perceived clinical utility. While HPs recognise the importance of pre-test counseling for informed decision-making, they reported various barriers to the provision of adequate pre-test counseling. Further research on the post-test counseling experiences of HPs is recommended in light of comparatively limited research in this area. Addressing barriers in clinical practice and increasing consistency across and access to clinical guidelines and education resources may support HPs in supporting pregnant people and couples’ reproductive autonomy.

## Supplementary Information

Below is the link to the electronic supplementary material.

Supplementary file1 (DOCX 284 KB)

## Data Availability

No datasets were generated or analysed during the current study.
