# Supplementary material for: Healthcare professionals’ perspectives on and experiences with non-invasive prenatal testing: a systematic review
Source: Hum Genet. 2025 Apr 9;144(4):343–74. doi: 10.1007/s00439-025-02736-y (PMC12003526; doi:10.1007/s00439-025-02736-y)
Supplement: Supplementary file 1 — Supplementary file1 (DOCX 284 KB) [file 439_2025_2736_MOESM1_ESM.docx]

# **Supplement 1. Search terms**

|  | **MEDLINE** | **Embase** | **SCOPUS** | **Web of Science** |
| --- | --- | --- | --- | --- |
| 1 | Noninvasive Prenatal Testing/ | noninvasive prenatal testing/ | TITLE-ABS-KEY ( "non-invasive prenatal test*" OR "non-invasive prenatal screen*" OR "non-invasive prenatal diagnos*" OR "noninvasive prenatal test*" OR "noninvasive prenatal screen*" OR "noninvasive prenatal diagnos*" OR "cfdna" OR "cffdna" | "non-invasive prenatal test*" OR "non-invasive prenatal screen*" OR "non-invasive prenatal diagnos*" OR "noninvasive prenatal test*" OR "noninvasive prenatal screen*" OR "noninvasive prenatal diagnos*" OR "cfdna" OR "cffdna" (All Fields) |
| 2 | empirical research/ or qualitative research/ | qualitative research/ | TITLE-ABS-KEY ( qualitative OR interview* OR "focus group*" OR empirical OR survey* OR questionnaire* ) | qualitative OR interview* OR "focus group*" OR empirical OR survey* OR questionnaire* (All Fields) |
| 3 | Interview/ | empirical research/ | ( LIMIT-TO ( LANGUAGE , "english" ) ) | English (Languages) |
| 4 | Focus Groups/ | structured interview/ or interview/ or semi structured interview/ or unstructured interview/ | 1 and 2 and 3 | 1 and 2 and 3 |
| 5 | "Surveys and Questionnaires"/ | health survey/ |  |  |
| 6 | 2 or 3 or 4 or 5 | health care survey/ |  |  |
| 7 | 1 and 6 | questionnaire/ |  |  |
| 8 | (Non-invasive prenatal test* or non-invasive prenatal screen* or non-invasive prenatal diagnos* or cfDNA or cffDNA).mp. | 2 or 3 or 4 or 5 or 6 or 7 |  |  |
| 9 | (qualitative or interview* or focus group* or empirical or survey* or questionnaire*).mp. | 1 and 8 |  |  |
| 10 | 8 and 9 | (Non-invasive prenatal test* or non-invasive prenatal screen* or non-invasive prenatal diagnos* or cfDNA or cffDNA).mp. |  |  |
| 11 | 7 or 10 | (qualitative or interview* or focus group* or empirical or survey* or questionnaire*).mp. |  |  |
| 12 | limit 11 to english | 10 and 11 |  |  |
| 13 |  | 9 or 12 |  |  |
| 14 |  | limit 13 to english |  |  |
